# Immune Cell Engagers and “Masking Immunotherapy” for Solid Tumors: Focus on Head and Neck Cancers

**DOI:** 10.3390/cancers18203401

**Published:** 2026-10-21

**Authors:** Giuseppe Nardo, Deborah Lenoci, Marta Lucchetta, Lisa Licitra, Loris De Cecco, Stefano Cavalieri

**Affiliations:** 1 Head and Neck Medical Oncology Department, Fondazione IRCCS Istituto Nazionale dei Tumori, 20133 Milan, Italy; 2 Integrated Biology of Rare Tumors, Department of Experimental Oncology, Fondazione IRCCS Istituto Nazionale dei Tumori, 20133 Milan, Italy; 3 Department of Oncology and Hemato-Oncology, University of Milan, 20122 Milan, Italy

**Keywords:** immune-cell engagers, ICE, T-cell engagers, TCE, gamma-delta TCE, NKCE, phagocytic-engager, HNSCCs, head and neck cancers, masking immunotherapy

## Abstract

Head and neck squamous cell carcinomas (HNSCCs) are challenging pathologies to treat with immune checkpoint inhibitors (ICIs), as only a minority of patients achieve durable clinical benefit. The highly immunosuppressive tumor microenvironment (TME), the frequent loss of antigen presentation, the marked spatial heterogeneity, and the anatomical proximity of tumors to critical structures limit the efficacy and safety of current immunotherapeutic approaches. Immune cell engagers (ICEs) have emerged as promising strategies to redirect endogenous immune cells toward tumor targets. While conventional surface-antigen-directed engagers can bypass major histocompatibility complex (MHC)-restricted antigen presentation, peptide–HLA-directed constructs remain dependent on antigen processing, peptide presentation, and the appropriate HLA allele. More recently, tumor-restricted activation technologies, collectively referred to as “masking immunotherapy”, have been developed to preferentially restore target and/or immune-cell engagement under TME-associated conditions, such as increased protease activity. These approaches have demonstrated enhanced tumor-preferential activity and/or improved tolerability in preclinical models; however, the extent to which these properties translate into clinically meaningful tumor selectivity and an improved therapeutic index remains under investigation. In this review, based on the authors’ comprehensive literature knowledge and targeted searches, we critically examine the biological rationale, the engineering principles, the current preclinical evidence, and the early clinical development of ICEs with particular emphasis on their potential application in HNSCC. While early clinical studies have demonstrated acceptable safety profiles, clinical activity has generally been poor. We discuss the major barriers that limit clinical translation and we highlight emerging strategies aimed at overcoming these limitations. We believe that successful clinical development of ICEs in HNSCC will likely depend not only on the identification of optimal target antigens, but also on spatially controlled immune activation, integration with TME-modulating approaches, and incorporation of dynamic biomarkers into future clinical trial design.

## Simple Summary

Head and neck squamous cell carcinomas (HNSCCs) are difficult to treat with current therapeutic options because of anatomical and biological limits. Immune cell engagers (ICEs) are a new generation of engineered antibodies designed to redirect the patient’s own immune cells toward cancer cells, offering the potential to overcome several mechanisms of immune escape. More recently, innovative approaches such as conditionally activated or “masked” platforms have been designed to preferentially restore immune-engaging activity within the TME. Although preclinical studies support the potential of these approaches to reduce systemic immune activation and on-target, off-tumor toxicity, their degree of tumor selectivity and safety benefit in patients remains to be established. In this review, we summarize the current landscape of ICEs in HNSCC and discuss their biological rationale, available preclinical and clinical evidence, and the main barriers limiting their clinical translation. We also highlight emerging engineering strategies and future directions that may help improve the efficacy and safety of these promising immunotherapeutic platforms.

## 1. Introduction

HNSCCs comprise a heterogeneous group of malignancies that arise from the mucosal epithelium of the upper aerodigestive tract and display striking biological diversity according to their etiologic drivers and anatomic sites. This heterogeneity profoundly influences the TME and ultimately dictates responsiveness to immunotherapeutic approaches. In this context, emerging translational evidence further suggests that these immune dynamics are not static but evolve under therapeutic pressure, with longitudinal remodeling of antigen expression, immune-cell spatial distribution, and cytokine gradients. Such plasticity has direct implications for the durability of immune-based therapies and underscores the importance of adaptive treatment strategies, real-time biomarker monitoring, and rational combination approaches aimed at sustaining effective antitumor immunity over time [1].

Among the different HNSCC subtypes, human papillomavirus (HPV)-associated oropharyngeal carcinomas frequently harbor a highly inflamed milieu, enriched in tumor-infiltrating lymphocytes (TILs) and characterized by elevated interferon-γ (IFN-γ)-related gene signatures. Despite this pre-existing immune activation, many HPV-positive tumors exhibit adaptive immune resistance, mediated by up-regulation of the programmed death-1 (PD-1)/programmed death-ligand 1 (PD-L1) axis within TME. This duality explains the favorable clinical responses to PD-1 blockade observed in this subgroup [2].

In contrast, Epstein–Barr virus (EBV)-driven nasopharyngeal carcinoma represents a virally induced malignancy with unique immunobiological attributes. The presence of latent and lytic viral antigens provides defined targets for immune recognition, while the lymphoid-rich stroma and extensive plasma-cell infiltrates reflect an active immune engagement. Such features make EBV-related disease an appealing context for T cells-redirecting and antigen-specific immunotherapies [3].

Tobacco- and alcohol-related HNSCCs are typically HPV-negative and genomically complex, with high tumor mutational burden (TMB) but limited immune infiltration. Active smokers display diminished cluster differentiation (CD) 8 positive T cells density and reduced interferon-α/γ pathway signaling, together with suppression of the CXC motif chemokine ligand (CXCL)9/10/11-CXC motif chemokine receptor 3 (CXCR3) axis, which orchestrates effector-cell trafficking. These features suggest a “cold” immunologic phenotype in which both recruitment and activation of cytotoxic lymphocytes are impaired. The resulting immunosuppressive TME may explain the relatively modest responses of HPV-negative tumors to immune checkpoint blockade (ICB) and highlights the need for therapeutic modalities capable of reshaping local immunity [4,5,6].

Beyond viral and chemical carcinogenesis, environmental exposures, such as wood dust, nickel, or formaldehyde, contribute to sinonasal and other site-specific malignancies with distinct immunopathologic profiles. These tumors often exhibit low TMB and unique stromal and myeloid architectures, reinforcing the concept that anatomic and etiologic context must guide the design of immune interventions [7,8].

Over the past decade, ICIs have confirmed the centrality of the immune milieu in HNSCC pathogenesis. Agents targeting PD-1 or PD-L1, such as pembrolizumab, nivolumab, or atezolizumab, usually produce durable clinical benefit in a subset of patients across HPV statuses. Molecular analyses consistently demonstrate that PD-L1 expression and IFN-γ-responsive gene signatures correlate with therapeutic benefit, reinforcing the link between pre-existing inflammation and checkpoint responsiveness [9,10].

Taken together, these observations portray HNSCCs not as a single disease but as a spectrum of immune ecosystems shaped by viral, environmental, and behavioral factors. This immunologic diversity implies that therapeutic success in HNSCCs is less likely to depend on universal immune activation and more on approaches capable of selectively engaging or reprogramming the dominant immune context present within each etiologic subset. Understanding this spectrum is critical for the rational integration of next-generation immunotherapies.

In this review, based on the authors’ comprehensive literature knowledge and targeted searches of PubMed/MEDLINE and ClinicalTrials.gov, we critically examine the biological rationale, engineering principles, current preclinical evidence, and early clinical development of ICEs, with particular emphasis on their potential application in HNSCC. The literature and clinical-trial information were last updated in September 2026. Given the narrative nature of this review, study selection was not based on a predefined systematic-review protocol.

## 2. The HNSCCs’ TME: Barriers and Opportunities for ICEs

HNSCCs’ TME represents a dynamic and immunologically complex ecosystem that exerts a profound influence on disease progression and therapeutic responsiveness [11]. It is composed of a diverse array of cellular and molecular elements, immune and stromal cells, vascular and lymphatic networks, extracellular matrix components, and soluble mediators, that collectively shape the balance between antitumor immunity and immune suppression. In this setting, ICEs offer a unique opportunity to bypass upstream defects in antigen presentation and immune priming by enforcing direct cytotoxic synapses [12].

At baseline, most HNSCCs display evidence of chronic inflammation intertwined with immune evasion. In this context, TILs coexist with regulatory T lymphocytes (Tregs), myeloid-derived suppressor cells (MDSCs), and tumor-associated macrophages (TAMs), three of the most abundant TME cellular populations. They secrete inhibitory cytokines, such as transforming growth factor-β (TGF-β) and interleukin-10 (IL-10), produce metabolic suppressors, including arginase and indoleamine 2,3-dioxygenase (IDO), and express exhaustion markers, like PD-L1, T-cell immunoglobulin domain and mucin domain 3 (TIM-3), and V-domain Ig Suppressor of T cell Activation (VISTA) [13]. Through these mechanisms, they blunt effector T-cell proliferation and cytotoxicity, suppress antigen presentation, and dampen type-I interferon responses.

Beyond soluble mediators, these myeloid and regulatory populations actively participate in tumor remodeling processes, driving epithelial–mesenchymal transition, promoting angiogenesis, and facilitating metastatic dissemination. The resulting TME is not merely immunologically inert, but structurally and metabolically remodeled to exclude effector cells and support immune escape. High densities of TAMs and MDSCs correlate with poor prognosis, reduced response to immunotherapy, and increased therapy resistance.

Recent advances in spatial transcriptomics and digital pathology have revealed that the spatial distribution of immune populations within HNSCCs tissues holds major prognostic and functional significance [14]. Tumors with a “hot” architecture, where CD8^+^ T cells and dendritic cells (DCs) penetrate in the tumor cores and closely interact with malignant cells, exhibit higher expression of interferon-stimulated genes and improved clinical outcomes. Conversely, “immune-excluded” or “immune-desert” phenotypes, characterized by peritumoral localization, display resistance to ICB. Quantitative spatial metrics, such as G-cross functions derived from hematoxylin–eosin (H&E) and multiplex immunofluorescence imaging, now provide objective correlates of immune infiltration, activation status, and radiosensitivity. Such measurements reinforce that the geometry of immune engagement within the tumor is as important as the overall immune-cell density when predicting therapy response.

Within this landscape, tertiary lymphoid structures (TLSs) have emerged as key immune hubs. These ectopic lymphoid aggregates, enriched in mature DCs and B cells, function as local sites of antigen presentation and T-cell priming. In HNSCCs, the presence and maturation state of TLSs strongly correlate with improved survival and superior responses to ICB [15]. Importantly, TLS-related transcriptional signatures may outperform PD-L1 combined positive score (CPS) as predictive biomarkers. This insight has encouraged the incorporation of TLS assessment and spatial biomarker endpoints into the design of ongoing immunotherapy trials.

The pre-existing inflammatory state and molecular characteristics of a tumor further influence its immune responsiveness. An IFN-γ-responsive transcriptional profile has been associated with increased sensitivity to PD-1 blockade. In addition, within HPV-negative HNSCC, an APOBEC-enriched molecular subset has been associated with increased T cells inflammation and immune-checkpoint expression, suggesting that APOBEC-driven mutagenesis may identify a genomically defined subgroup with enhanced immune priming [10]. In contrast, active smoking is associated with broad suppression of IFN pathways, reduced CXCL9/10/11 expression, and diminished CD8^+^ T cells infiltration [16]. These findings imply that ICE deployment may benefit from TME “conditioning” strategies, such as radiation, oncolytic viruses, or targeted cytokine modulation, to prime the environment for immune engagement. Moreover, lifestyle factors like smoking cessation during treatment might augment such immunologic reprogramming by restoring interferon responsiveness.

From a therapeutic perspective, this complex immunobiology poses both obstacles and opportunities for ICEs. On one hand, the physical barriers of dense desmoplastic stroma, irregular vasculature, and hypoxia can restrict antibody diffusion and effector-cell trafficking, potentially limiting the intratumoral concentration of ICEs. On the other hand, the abundance of immunosuppressive cellular populations also provides new targets for restoring immune system. For instance, strategies that redirect resident macrophages or neutrophils toward antibody-dependent cellular cytotoxicity (ADCC) or phagocytosis could convert immunosuppressive compartments into effector allies. Similarly, leveraging TLS formation or local antigen presentation to recruit and activate bystander T cells could synergize with ICEs.

## 3. ICEs: Concepts, Classes, and Design Principles

Over the past decade, ICIs have reshaped the therapeutic landscape of solid tumors, including HNSCCs [17,18]. However, only a subset of patients experiences meaningful benefit from these agents, underscoring the need for next-generation immunotherapies that can actively recruit and bridge immune cells toward malignant targets, even within immunologically “cold” tumors [19,20]. In this context ICEs have emerged as promising immunotherapeutic agents designed to redirect non-tumor committed immune cells to tumor cells, thereby inducing direct cytotoxicity and immune activation [21,22]. Collectively, these features position ICEs as a flexible immunotherapeutic framework that can be adapted across immune contexts, effector cell types, and tumor architectures.

### 3.1. Fundamental Concepts

ICEs function as molecular matchmakers: by simultaneously binding a tumor-associated antigen (TAA) and an activating receptor on an immune cell, they bring the two into close proximity and trigger the formation of an immunological synapse [23]. For conventional surface-antigen-directed engagers, this mechanism can bypass classical MHC-restricted antigen recognition and does not require conventional co-stimulation. However, this property does not apply to peptide–HLA-directed TCR-based or TCR-mimic engagers, whose activity requires intracellular antigen processing, presentation of the cognate peptide, and expression of the appropriate HLA allele [10,22,24]. The resulting immune activation leads to targeted killing of tumor cells while theoretically sparing healthy tissues lacking the specific antigen. Thanks to this innovative design, also immune cells that are not tumor-specific become activated against the tumor, thereby amplifying immune system activation. This enforced proximity-driven activation allows immune engagement to occur even in settings where endogenous antigen recognition and priming are insufficient to initiate an effective antitumor response [10,19].

### 3.2. Classes of ICEs

**Figure 1:**
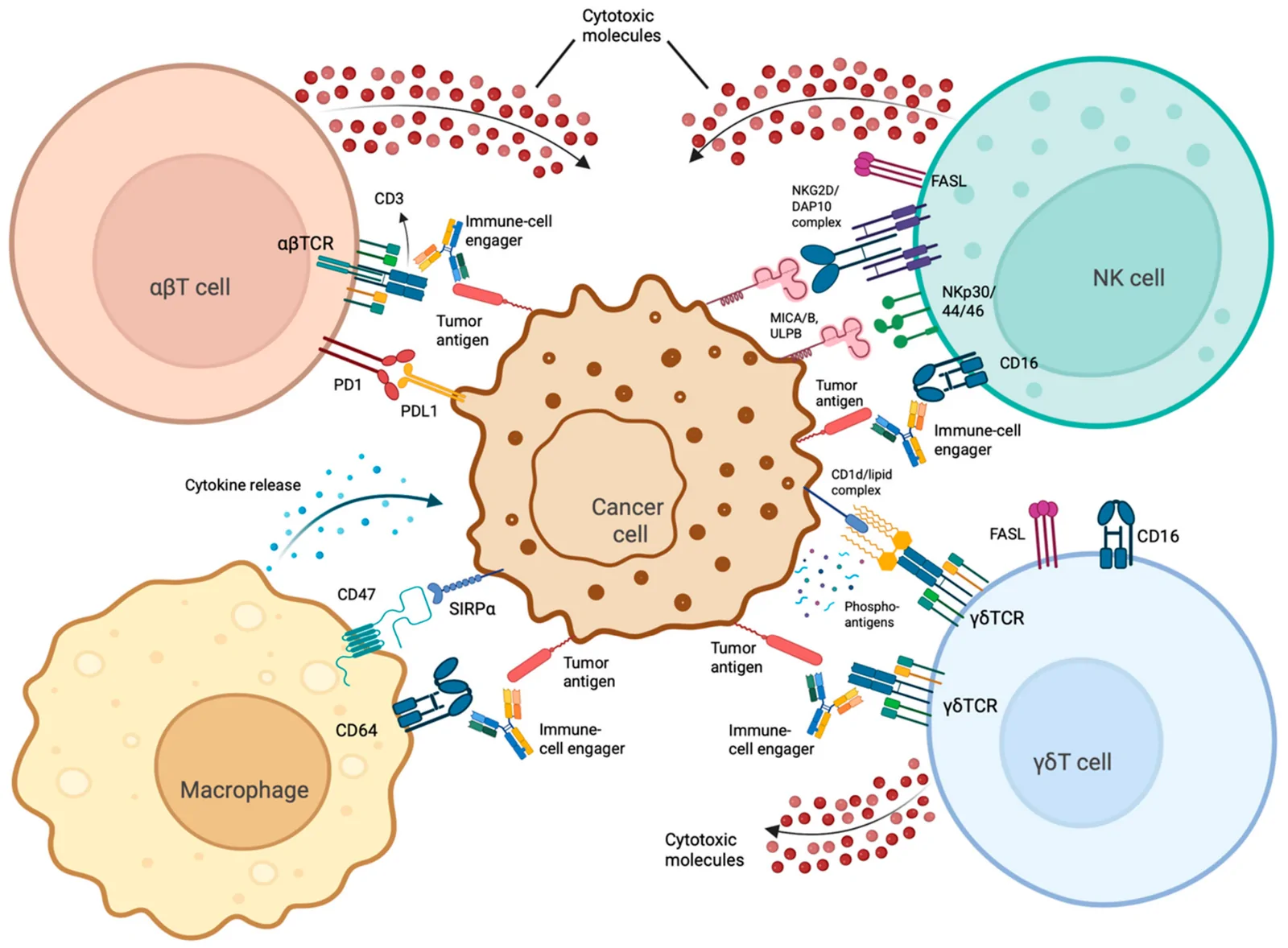
The figure illustrates a cancer cell interacting with four types of ICEs. T-cell engagers (**upper left**) typically link tumor antigens to CD3 on αβ T cells, promoting cytotoxic synapse formation and release of cytotoxic molecules. The PD1/PDL1 binding is one of the main pathways that modulates immune effector activity, and even if it is represented only on this cell, it could be present on all immune cells. NK-cell engagers (**upper right**) can recruit NK cells through activating receptors such as CD16, NKp30, or NKG2D, whereas γδT-cell engagers exploit γδ T-cell-associated recognition pathways involving CD1d/lipid complex and phosphoantigens, together with NK cells activating pathways. Myeloid-cell engagers may promote macrophage activation through activating receptors such as CD64 and phagocytosis through CD47–SIRPα binding. Legend: TCR, T-cell receptor; CD, cluster of differentiation; PD1/PDL1, programmed death 1/programmed death ligand 1; NKG2D, natural killer group 2 member D; SIRPα, signal regulatory protein alpha; MICA/B, MHC class I polypeptide-related sequence A or B; ULBP, UL16-binding protein; FASL, Fas ligand.

Several ICE modalities have been developed, as summarized below and illustrated in Figure 1.

(a) T-cell engagers (TCEs): TCEs are the most established class of ICEs and typically consist of bispecific antibodies or antibody fragments that link CD3 on T cells with a TAA on tumor cells. Engagement of CD3 induces T cells activation, degranulation, and perforin/granzyme-mediated cytotoxicity against antigen-bearing targets [25]. The paradigm-shifting success of the CD19×CD3 bispecific blinatumomab in acute lymphoblastic leukemia validated this concept in hematologic malignancies [26]. In solid tumors, the challenge is harder: TCEs must function within the immunosuppressive TME, where effector cells may be excluded, exhausted, or metabolically suppressed. This constraint has driven the evolution of TCEs design toward formats that better accommodate the spatial, metabolic, and immunosuppressive features of solid tumors.
(b) Natural killer cell engagers (NKCEs): NKCEs bridge TAAs to activating receptors on NK cells, such as CD16A (FcγRIIIa), NKp30, or NKG2D, thereby triggering ADCC or innate cytotoxic programs [27]. NKCEs exploit the innate immune system’s ability to recognize and destroy abnormal cells without prior sensitization [28]. In the context of HNSCC, NK cells represent an attractive effector population, although their antitumor activity can be impaired by the immunosuppressive TME. Indeed, tumor-infiltrating NK cells from patients with HNSCC exhibit an altered receptor profile and reduced ADCC capacity. NKCEs may therefore provide a strategy to redirect and enhance endogenous NK cells cytotoxicity against tumor cells [27,29].
(c) γδ T-cell engagers (γδTCEs): γδT cells are unconventional lymphocytes that recognize stress-induced ligands and phosphoantigens independently of classical antigen presentation [30]. γδTCEs connect tumor antigens to γδ T-cell receptor (TCR) or their regulatory butyrophilin (BTN) 2A1/3A1 complexes, activating cytotoxic responses that bridge innate and adaptive immunity [31,32]. By exploiting stress-sensing mechanisms rather than classical antigen presentation, γδTCEs engage cytotoxic immunity in tumors that evade conventional T cells surveillance [30]. Their MHC-independent recognition provides a compelling rationale for targeting tumors with impaired antigen presentation; however, their ability to maintain antitumor activity in highly immunosuppressive HNSCC has not yet been defined.
(d) Phagocytic and dendritic cell engagers: Emerging ICE architectures now extend beyond cytotoxic lymphocytes to include myeloid effectors [33]. These constructs promote phagocytosis or antigen uptake by macrophages and dendritic cells, thereby facilitating antigen cross-presentation and subsequent epitope spreading [34]. Incorporating myeloid engagement into ICE platforms expands the therapeutic scope beyond direct cytotoxicity, enabling immune amplification through antigen presentation and clonal diversification.

### 3.3. Design Principles and Challenges in Solid Tumors

Translating the ICE concept into solid tumors has required iterative innovation in molecular design [20,35]. The dense, protease-rich, and hypoxic TME imposes several constraints that differ from hematologic settings [36]. Key design imperatives include:

- **Enhanced tumor selectivity:** To minimize on-target, off-tumor toxicity in normal epithelial tissues, especially critical in the anatomically delicate head and neck region where vital structures are in close proximity to tumor sites [37].
- **Optimized pharmacokinetics and bioavailability:** Classical bispecific TCEs (BiTEs) are small single-chain variable fragments (scFvs) with short half-lives that require continuous infusion; novel half-life extended (HLE) designs incorporate Fc domains or albumin-binding modules to prolong circulation and allow intermittent dosing [38].
- **Improved tumor penetration:** Multivalent and modular bispecific architectures enable optimization of molecular size, valency, avidity, and target engagement, which may influence tumor penetration and pharmacokinetic properties [39,40].
- **Resistance to immunosuppression:** Combining TCE activity with intrinsic ICB or cytokine delivery can maintain T-cell activation despite inhibitory cytokines or metabolic stressors [41].
- **Adaptation to antigen heterogeneity:** Bispecific or multispecific targeting of different TAAs reduces the risk of antigen-loss variants, a frequent mechanism of resistance in solid tumors [42,43].

Together, these design principles underscore that effective ICE development in solid tumors requires simultaneous optimization of selectivity, persistence, and microenvironmental fitness. The rapid evolution of computational antibody engineering and synthetic biology has further enabled logic-gated and conditionally activatable designs [44]. Advances in computational antibody engineering and synthetic biology have enabled increasingly sophisticated strategies to improve tumor selectivity. In engineered cellular therapies, combinatorial antigen-sensing circuits can implement AND- or NOT-gate logic to discriminate tumor cells from normal tissues expressing individual target antigens [45,46]. Analogous principles of conditional activation are being pursued in soluble T-cell engagers through approaches such as protease-dependent masking and tumor-restricted activation, as discussed below.

### 3.4. Safety Considerations and Toxicity Management

The toxicity profile of ICEs depends substantially on the effector-cell pathway engaged. Cytokine-release syndrome (CRS) is commonly associated with TCE, whereas immune effector cell-associated neurotoxicity syndrome (ICANS) has also been reported with some T cells-redirecting platforms, although its frequency and severity vary across agents. By contrast, the safety profiles of NKCE, γδTCE, and myeloid-cell engagers are clinically less characterized and should not be assumed to reproduce the toxicity spectrum of conventional CD3-directed TCEs. Early clinical experience with NKCE, for example, has identified infusion-related reactions and cytokine-mediated events, but CRS and ICANS have not emerged as uniformly dominant class toxicities. For γδT-cell and myeloid-cell engager platforms, clinical experience remains too limited to define a characteristic toxicity profile [47,48,49]. Regardless of engager class, local inflammatory toxicity may be particularly relevant in HNSCC. The clinical consequences of local immune activation may be amplified by anatomy: inflammatory swelling, tumor necrosis, or bleeding occurring adjacent to the upper airway and swallowing structures may become clinically significant even when systemic toxicity remains manageable [50]. Therefore, the therapeutic-index problem may be particularly relevant in HNSCCs, and the safety management becomes not merely supportive but an integral component of therapeutic efficacy. Many candidate targets for immune redirection, including EGFR and other epithelial antigens, are tumor-associated rather than strictly tumor-specific, creating a potential risk of on-target, off-tumor activity in normal tissues. Thus, for HNSCC, therapeutic index should be considered not only in terms of systemic cytokine exposure but also in terms of the spatial distribution of immune activation.

Modern clinical protocols incorporate step-up dosing, premedication with corticosteroids or IL-6 inhibitors, and rapid escalation contingency plans coordinated with emergency and critical-care teams. Equally vital is clinician education, ensuring early recognition and standardized management pathways [51].

Conditionally activated TCEs are designed to preferentially restore engager activity under TME-associated conditions, with the potential to mitigate systemic on-target, off-tumor toxicity. However, the magnitude of tumor selectivity and any resulting clinical safety benefit is to be defined [52]. In anatomically confined head and neck tumors, treatment-related inflammation, edema, or bleeding may have clinically important consequences; therefore, careful airway assessment and multidisciplinary management should be considered when developing highly inflammatory immune-redirecting strategies. Combination regimens, particularly TCE + PD-1 blockade, demand vigilant escalation strategies guided by real-time cytokine panels and spatial pharmacodynamic data.

### 3.5. Relevance to Head and Neck Oncology

HNSCCs provide a compelling clinical context for ICE development. Moreover, the coexistence of immunologically “hot” (HPV-positive) and “cold” (HPV-negative, smoking-related) phenotypes allows exploration of ICE performance across diverse immune ecosystems [4]. The frequent downregulation of MHC, the presence of viral or tumor-specific antigens, and the unmet need in recurrent/metastatic disease make HNSCCs an ideal proving ground for these modular immunotherapies [24].

In summary, ICEs represent a versatile and rapidly expanding class of biologics that can redirect innate and adaptive immune responses with remarkable precision. Surface-antigen-directed engagers can bypass conventional MHC-restricted antigen presentation, potentially addressing one mechanism of immune escape in HNSCC. By contrast, peptide–HLA-directed constructs expand the targetable antigen repertoire to intracellular proteins but remain dependent on antigen processing, peptide presentation, and HLA compatibility. Continued refinement of their molecular architectures, delivery strategies, and combinatorial use with immune conditioning approaches is likely to define the next frontier in the treatment of these complex malignancies.

## 4. TCEs for HNSCCs: Modalities and Innovations

Currently, within solid tumors only two TCEs have been approved in clinical practice: Tebentafusp, a gp100 peptide-HLA-A*02:01 directed TCR/CD3 bispecific protein for uveal melanoma [53]; and Tarlatamab, an HLE DLL3-CD3 TCE for patients with small-cell lung cancer (SCLC) after progression to standard of care platinum chemotherapy [54,55]. Ongoing research is focused on refining TCE’s platform and extending their application in solid tumors.

### 4.1. Classical and Extended TCE Formats

TCEs constitute the most clinically advanced subset of ICEs. Canonical BiTEs, typically small single-chain variable fragments (scFvs) linking CD3 on T cells with a tumor-associated antigen (TAA), mediate potent MHC-independent cytotoxicity. Their minimal architecture allows rapid synapse formation between T cells and tumor targets, resulting in perforin- and granzyme-dependent killing. However, in solid tumors early-generation constructs face severe hurdles: short serum half-life, poor tumor penetration, and functional suppression of infiltrating T cells within the TME. These limitations underscore that synapse potency alone is insufficient unless it is accompanied by pharmacokinetic and TME-aware engineering.

To address these barriers, next-generation molecules incorporate HLE features, such as Fc domains, albumin-binding modules, or polymer conjugation, which prolong systemic exposure and enable more flexible dosing schedules [38]. Multivalent or trivalent designs, for example DOCK-AND-LOCK constructs, enhance avidity and tumor retention, while mitigating the need for continuous infusion [56]. In HNSCC, HLE-TCEs may offer practical pharmacokinetic advantages by prolonging systemic exposure and reducing the need for continuous infusion. However, improved pharmacokinetics alone should not be interpreted as evidence of reduced CRS, which remains dependent on construct design, target biology, dose, and treatment schedule.

Preclinical studies further indicate that TCEs can synergize with PD-1/PD-L1 blockade, as checkpoint inhibition reinvigorates exhausted T cells engaged by bispecifics [41].

### 4.2. Co-Stimulation and Logic Gating

One limitation of conventional TCEs is the absence of a co-stimulatory “signal 2,” which can lead to suboptimal activation or premature exhaustion of effector T cells. To address this, Simultaneous Multiple Interaction T-cell Engagers (SMITEs) or dual-module constructs combine CD3 redirection (signal 1) with a co-stimulatory domain targeting CD28, 4-1BB (CD137), or OX40 (CD134) (signal 2) [57]. These strategies aim to enhance T cells activation through coordinated signaling; however, the presence of two signaling modules does not necessarily establish strict AND-gate behavior or prevent off-tumor activation.

Other platforms have been investigated in experimental soluble TCE architectures, including split-CD3 and affinity-tuned dual-antigen-targeting constructs, in which productive T-cell activation preferentially occurs when the required tumor antigens are co-expressed. Such AND-like behavior has been demonstrated primarily in preclinical models, and its capacity to improve clinical tumor selectivity or reduce on-target, off-tumor toxicity remains unproven [58]. By contrast, inhibitory NOT-gating and programmable antigen-sensing circuits have been developed predominantly in engineered cellular therapies, including CAR-T cells [59]. Therefore, these mechanisms should not be considered established properties of conventional soluble engagers.

### 4.3. Targeting Induced T-Cell Activating Nanobodies (TITAN)

The TITAN platform represents a CD8-guided TCE technology designed to improve the therapeutic index of TCEs by preferentially recruiting CD8^+^ cytotoxic T cells rather than broadly activating T cells populations. By incorporating binding domains against both TCR (or CD3) and the CD8 co-receptor, in addition to tumor-targeting moieties, TITAN-based molecules are designed to limit CD4^+^ T cells engagement and potentially reduce cytokine production while maintaining cytotoxic activity. However, whether CD8-selective engagement translates into a clinically meaningful reduction in CRS or an improved therapeutic index remains to be established [60]. This CD8-focused engagement strategy may be particularly advantageous in HNSCC, where limiting excessive cytokine release is essential to preserve airway safety while maintaining cytotoxic efficacy. Early clinical development has been initiated with AZD5492, a CD20-directed TITAN construct investigated in a Phase 1/2 trial in B cells malignancies (NCT06542250) [61].

### 4.4. Checkpoint-Inhibitory TCEs (CITEs)

CITEs represent an elegant convergence of TCEs and ICB within a single molecule. CITEs combine a CD3-redirecting arm with a second domain that neutralizes inhibitory interactions, most commonly PD-1/PD-L1 or Cytotoxic T-Lymphocyte-Associated protein 4 (CTLA-4). Some constructs incorporate enzymatic modules capable of removing suppressive glycans or adenosine-generating ectoenzymes, thereby reversing local immune paralysis at the immunologic synapse.

Preclinical studies have shown that incorporation of checkpoint-inhibitory or glycan-modifying modules into a TCE can enhance T cells-mediated tumor-cell killing compared with the corresponding conventional BiTE scaffold [62]. By integrating checkpoint modulation with T cells redirection, CITEs are designed to enhance antitumor effector functions at the immune synapse and may reduce the need for separate systemic checkpoint blockade. However, whether this design improves tissue selectivity or reduces systemic immune-related toxicity remains under investigation. In HNSCCs, where PD-L1 is often co-expressed with TAAs, such as epidermal growth factor receptor (EGFR) or epithelial cell adhesion molecule (EpCAM), this dual mechanism could simultaneously redirect cytotoxicity and dismantle inhibitory signaling, a particularly valuable feature for tumors refractory to systemic PD-1 inhibitors. Such spatially confined checkpoint modulation may be especially relevant in HNSCC, where systemic immune-related toxicity often limits the intensification of immunotherapy combinations.

### 4.5. Localized Delivery: Secreted or Tumor-Expressed BiTEs

Given the anatomical sensitivity of head and neck sites, strategies that confine immune activation to the tumor bed are gaining traction. Localized TCE delivery can be achieved through engineered immune or stromal cells, viral vectors, or oncolytic platforms capable of secreting bispecifics directly within tumors. For example, CAR-T cells engineered to secrete BiTEs, so-called “armored CARs”, extend cytotoxic reach beyond the engineered population, recruiting bystander T cells and amplifying immune pressure locally while minimizing systemic toxicity [63].

Similarly, oncolytic viruses provide a dual benefit: they lyse tumor cells, releasing neoantigens, and deliver payloads encoding TCEs or immune agonists. In preclinical HNSCC models, tumor-tropic vaccinia and adenoviral vectors expressing immune engagers induce local cytokine release, DC priming, and vascular normalization, thereby improving drug access and promoting a more inflamed microenvironment [64]. This paradigm exemplifies therapeutic complementarity, coupling TCE-mediated cytotoxicity with oncolytic reprogramming of the TME. By decoupling systemic exposure from intratumoral immune activation, localized TCE delivery offers a pragmatic solution to the narrow therapeutic window characteristic of head and neck malignancies.

### 4.6. Masked or Protease-Activatable TCEs

One of the most innovative developments is the advent of “masked” or protease activatable TCEs, designed to remain inactivate or attenuated in circulation and to become functional preferentially within the proteolytic milieu of the tumor. These constructs incorporate a peptide or antibody-derived masking domain that limits target engagement until cleaved by tumor-enriched proteases such as matrix metalloproteinases (MMPs) or cathepsins. Upon local activation, the unmasked molecule engages CD3 and triggers T cells cytotoxicity preferentially within the TME [52]. However, activation is not necessarily exclusive to malignant tissues, because relevant proteases may also be present in inflamed or remodeling normal tissues. This approach is intended to improve the therapeutic index of systemic TCEs by reducing premature target engagement and potentially limiting on-target, off-tumor toxicity. Such advantages have been supported by preclinical findings for selected constructs, but a consistent improvement in clinical safety or efficacy has not yet been established. Collectively, these tumor-activated modalities define a new generation of precision immunotherapies that combine the potency of T cells redirection with the safety of localized immune modulation. The success of these approaches will depend on accurate mapping of tumor-specific protease activity, which may vary across lesions and evolve under therapeutic pressure. Importantly, protease-dependent masking does not confer absolute tumor specificity. Proteases involved in mask cleavage may also be active in inflamed or remodeling normal tissues, potentially permitting off-tumor activation, while interpatient and interlesional heterogeneity in protease activity may result in variable intratumoral drug activation. Thus, the proposed therapeutic-index advantage depends on sufficient differential protease activity between tumor and normal tissues and requires validation for each individual construct.

### 4.7. Translational Outlook

Next-generation TCE platforms address distinct limitations of conventional T cells redirection in solid tumors. HLE TCEs improve pharmacokinetic properties and enable less frequent dosing, whereas multispecific and checkpoint-modulating constructs seek to combine T cells redirection with additional immunomodulatory functions. Conditionally activated TCEs instead aim to widen the therapeutic window by restricting T cellsengagement preferentially to the TME, an approach that may be particularly relevant for tumor-associated antigens also expressed in normal tissues. Other strategies, including CD8-selective, switchable, and logic-gated TCEs, provide additional mechanisms to control the specificity or intensity of immune-cell engagement, although their clinical development remains limited or at an early stage. Overall, these platforms provide complementary engineering solutions to the major barriers encountered by TCEs in solid tumors; however, robust clinical evidence demonstrating improved efficacy, safety, and durability in HNSCC remains limited.

Table 1 illustrates the different types of ICE platforms and their potential relevance in HNSCCs.

## 5. NKCEs in HNSCC

NK cells are a subset of innate immune cells that are involved in the eradication of transformed cells, like infected or tumoral cells. Unlike T cells, they recognize aberrant or stressed cells through the integration of activating and inhibitory receptor signals rather than antigen presentation via MHC molecules. This property allows them to overcome MHC I-downregulation resistance mechanism. Their ability to operate independently of MHC restriction positions them as ideal effectors for immune redirection strategies.

The engineering of NKCEs aims to harness and amplify these intrinsic properties by physically linking tumor antigens to NK-cell activation receptors. The most advanced formats involve bispecific or tandem antibodies (TandAbs) targeting CD16A (FcγRIIIa) on NK cells and a TAA such as EGFR. CD16A engagement triggers ADCC and degranulation, leading to potent tumor cell lysis. In preclinical models, EGFR/CD16A TandAbs have demonstrated robust NK-mediated killing of EGFR-positive tumor cells regardless of EGFR mutation status, establishing a mutation-agnostic therapeutic avenue for most HNSCCs that express EGFR at high levels [65].

Across a series of preclinical and early clinical studies, Miller et al. developed and characterized multiple formats of NKCEs; for instance, adding an IL-15 crosslinker (Trispecific Killer cell Engagers, TriKEs) leads to improved NK cell proliferation, higher cytotoxic granule release, better survival and delayed exhaustion in TME [66]. This point is crucial for solid tumors like HNSCC, where NK cell dysfunction is prominent. Several TriKEs constructs, such as B7-H3 or EGFR TriKE, showed strong antigen-specific NK-mediated killing of carcinoma cell lines, including HNSCCs, and enhanced NK cell degranulation and IFN-γ production upon engagement with HNSCC targets. A recurrent finding was that these molecules retain efficacy using primary NK cells from cancer patients, who often have impaired function; TriKEs restored cytotoxicity against patient-derived HNSCC tumor cells, and the IL-15 signal overcame NK cell hyporesponsiveness commonly observed in head-and-neck cancer patients. Earlier preclinical studies of B7-H3-targeted TriKEs demonstrated IL-15-mediated NK cell expansion, enhanced cytotoxicity against multiple B7-H3-positive solid-tumor models, and antitumor activity in vivo [67]. These findings suggest that IL-15 platforms may partially enhance the function of dysfunctional patient-derived NK cells. However, whether this approach can durably overcome the immunosuppression within the HNSCC TME remains to be defined.

Among the most clinically advanced NKCEs, AFM24, a bispecific EGFR×CD16A innate-cell engager, demonstrated potent EGFR-dependent cytotoxicity in preclinical solid-tumor models [65] and subsequently entered clinical evaluation in patients with advanced EGFR-positive solid tumors, where phase I data established clinical feasibility and a generally manageable safety profile [68]. These clinical data were not HNSCC-specific, and the clinical efficacy of AFM24 in HNSCC remains to be established.

Collectively, NKCEs provide an alternative strategy for immune-cell redirection in HNSCC. However, current clinical evidence is largely derived from other solid tumors, while NK cell dysfunction within the HNSCC TME remains an important biological barrier, and the clinical efficacy of NKCEs in this setting is to be defined.

## 6. Gamma-Delta T-Cell Engagers

γδT cells occupy an intermediate niche between innate and adaptive immunity, capable of recognizing stress-induced ligands and phosphoantigens independently of classical MHC presentation. Within this compartment, Vγ9Vδ2 T cells are the dominant circulating subset in humans and recognize transformed cells through conformational changes in BTN2A1/BTN3A1 complexes induced by intracellular phosphoantigens accumulation [31]. These cells mount rapid cytotoxic responses, secrete pro-inflammatory cytokines, and can even present antigen, thereby linking immediate tumor killing to longer-term immune priming [69]. Their MHC-independent recognition and rapid cytotoxic potential make γδT cells attractive effectors for solid tumors such as HNSCC, where defects in antigen presentation are frequent. However, their functional state can be shaped by chronic stimulation and by inhibitory signals within the TME. The development of γδTCEs seeks to exploit their unconventional recognition properties while enhancing resistance to immunosuppression [32].

Preclinical evidence supports the therapeutic potential of this approach: EGFR×Vγ9Vδ2 TCEs robustly activate γδT cells in vitro and in xenograft models, inducing tumor regression with limited toxicity [70]. These constructs elicit high levels of IFN-γ and TNF-α, enhance crosstalk with dendritic cells, and display minimal up-regulation of exhaustion markers such as PD-1, lymphocyte-activation gene (LAG)-3, or TIM-3. γδTCEs could also promote the activation of bystander immune populations, suggesting that their benefit may extend beyond direct cytotoxicity toward broad immune amplification. The MHC-independent activation of γδT cells may also mitigate selective pressure for antigen-presentation loss, a frequent resistance mechanism observed in recurrent and metastatic HNSCC.

Given the near-ubiquitous EGFR expression in HNSCCs and the frequent display of stress ligands in these tumors, γδTCEs provide a biologically congruent mechanism to overcome antigen-presentation deficits. Ex vivo expansion with IL-15 can enhance the cytotoxic phenotype of Vγ9Vδ2 T cells and increase expression of cytotoxic effector molecules, supporting cytokine-based strategies to enhance γδT cells activity [71]. Together, these attributes position γδTCEs as a complementary or alternative immune-engagement strategy for MHC-deficient, checkpoint-refractory HNSCCs.

## 7. Target Antigens in HNSCC for ICEs

A central determinant of ICE efficacy and safety is the careful selection of tumor-associated antigens that balance high tumor specificity with adequate surface expression to permit engagement. Antigen selection for ICE development in HNSCCs must integrate biological relevance with engineering feasibility, as the optimal target is defined not only by tumor expression but by how effectively it can be leveraged within conditional, spatially controlled immune engagement platforms. In HNSCCs, several targets stand out based on prevalence, biological function, and compatibility with logic-gated or masked formats:

(a) EGFR: Overexpressed in more than 90% of HNSCCs, EGFR remains the most validated antigen for immune engagement. However, it is also physiologically expressed in several epithelial and endothelial tissues. Consequently, EGFR-directed ICEs may carry a clinically relevant risk of on-target, off-tumor toxicity, providing a rationale for strategies aimed at increasing preferential activity within the TME [72]. CD16A-binding TandAbs and bispecifics directed against EGFR have demonstrated strong cytotoxicity and early clinical feasibility, and combination with existing anti-EGFR antibodies offers an additive ADCC advantage [52,65,66,67,68]. Despite EGFR clinical validation in HNSCC, EGFR-directed ICEs have not yet reproduced the clinical success of conventional anti-EGFR antibodies. This discrepancy likely reflects several factors, including the dependence of ICEs on immune-cell infiltration, antigen heterogeneity, the risk of on-target off-tumor toxicity, and the immunosuppressive HNSCC’s TME. These observations suggest that effective immune-cell redirection will require not only optimal target selection but also engineering strategies capable of overcoming the unique anatomical and biological barriers of HNSCC.
(b) CD44v6: A splice variant of the CD44 adhesion molecule, CD44v6 marks cancer stem-like populations and correlates with aggressiveness, metastasis, and therapy resistance. Although CD44v6 is frequently upregulated in HNSCC and enriched in tumor-associated stem-like populations, its expression is not tumor-exclusive and can also occur in normal epithelial tissues. This expression pattern supports its therapeutic relevance but also raises the possibility of on-target, off-tumor toxicity, providing a rationale for conditionally activated or logic-gated targeting strategies. Because stem-like cells may act as reservoirs for recurrence, engaging CD44v6 could facilitate dual targeting of both the bulk tumor and self-renewing subclones. Logic-gated or protease-masked configurations will likely be essential to minimize mucosal toxicity [73,74]. Targeting CD44v6 therefore aligns immune engagement with tumor hierarchy, potentially reducing relapse driven by stem-like compartments that are inadequately addressed by bulk-directed immunotherapies [75].
(c) B7-H3 (CD276): This immune-modulatory molecule is broadly overexpressed in HNSCCs and other solid tumors; however, its expression is not strictly tumor-specific and can be detected in selected normal tissues and non-malignant stromal compartments. Therefore, the potential for on-target, off-tumor effects should be considered when developing B7-H3-directed drugs [76]. B7-H3-directed CAR-T and ICE constructs (BiKEs, B7-H3/CD16) have demonstrated potent activity in preclinical studies, including squamous carcinoma models [64,66,77]. Its role in T-cell inhibition and angiogenesis suggests that B7-H3 targeting may simultaneously relieve immune suppression and deliver cytotoxicity, providing a dual mechanism of action relevant for HNSCCs [78,79]. This convergence of immune modulation and tumor-intrinsic biology positions B7-H3 as an attractive target for ICEs designed to exert both direct cytotoxic effects and TME modulation.
(d) Viral antigens: In virally driven subsets (HPV-positive or EBV-related tumors) viral oncogene products, such as E6 and E7, represent non-self antigens of high specificity [80]. TCR-mimic TCEs or bispecifics that recognize viral peptide-HLA complexes could achieve exquisite selectivity with minimal collateral toxicity [81]. Thus, viral peptide–HLA complexes represent highly tumor-selective targets for immune redirection, although their clinical utility remains dependent on antigen processing, HLA restriction, and adequate peptide presentation.
(e) Additional candidates: other antigens, such as mucin 1 (MUC1), trophoblast cell surface antigen 2 (TROP-2), carcinoembryonic antigen-related cell adhesion molecule 5 (CEACAM5), have been explored in other epithelial malignancies and show variable expression in HNSCCs [82,83,84]. Although less uniformly expressed, these antigens may gain relevance within multivalent or combinatorial ICE designs aimed at addressing interpatient and intratumoral heterogeneity.

Given the anatomical delicacy and mucosal proximity of HNSCC lesions, antigen selection must emphasize safety. Ideal candidates should permit masking or logic-gating to confine immune activation spatially, and multivalent designs may mitigate tumor heterogeneity while enabling the concurrent targeting of stem-like and differentiated compartments. In this context, EGFR, CD44v6, and B7-H3 stand as prioritized targets for near-term clinical translation.

## 8. Masking Immunotherapy in HNSCC

In this review, we use the term “masking immunotherapy” as a broad definition encompassing therapeutic platforms that are systemically inactive or attenuated and become pharmacologically active preferentially within the tumor microenvironment. Masking is an engineering strategy applicable to both conditionally activated ICEs, such as masked TCEs, and non-ICE platforms, including masked cytokines, ICIs, and antibody-drug conjugates (ADCs). These non-ICE modalities are discussed as proof-of-concept for tumor-restricted activation technologies that may inform the design of next-generation ICEs. Protease-dependent activation is particularly relevant to HNSCC, where MMPs, cathepsins, and other tumor-associated proteases may provide molecular switches for preferential intratumoral drug activation.

### 8.1. Masked Cytokines: IL-12 as an Archetype

Cytokines such as IL-12 have long been recognized for their ability to orchestrate type 1 immunity, activating CD8^+^ T cells and NK cells, enhancing IFN-γ production, and stimulating antigen presentation. Yet, the systemic administration of IL-12 has historically been plagued by severe toxicity, including CRS, hepatic inflammation, and hemodynamic instability. These limitations halted early clinical development despite compelling preclinical efficacy.

The advent of protease-activatable HLE IL-12 prodrugs, exemplified by XTX301, has redefined the therapeutic potential of this cytokine. XTX301 is engineered to attenuate IL-12 receptor engagement under systemic conditions and preferentially restore cytokine activity following proteolytic activation in the TME. However, the extent of systemic attenuation and tumor-preferential activation must be experimentally established. Within TME, MMPs and other tumor-enriched proteases cleave the linker sequence, promoting preferential release of active IL-12 within protease-rich tumor tissue. In murine and primate models, this strategy induced robust intratumoral IFN-γ signaling, increased CD8^+^ T-cell infiltration, and upregulated antigen-presentation machinery without eliciting systemic inflammatory toxicity [85]. By decoupling the powerful immunostimulatory effects of IL-12 from systemic exposure, masked cytokine strategies reposition this molecule from a historically toxic agent to a locally programmable amplifier of antitumor immunity.

In HNSCC, masked IL-12 therefore represents an exploratory TME-conditioning strategy whose ability to achieve spatially restricted immune activation and improve antitumor responses requires clinical validation.

### 8.2. BiTEs as an Example for Masking Immunotherapy

The success of masked cytokines has catalyzed parallel innovation in bispecific antibody engineering. Conventional TCEs often trigger dose-limiting cytokine release due to systemic CD3 engagement. Conditionally activated antibodies incorporate a peptide masking domain that limits target binding in normal tissues. This peptide mask is linked to the antibody via a cleavable linker that is sensitive to MMPs or cathepsins. Once the antibody arrives in the tumor site, the proteases cleave the linker and activate the antibody. This mechanism allows these drugs to deliver targeted therapy with reduced systemic immune activation and toxicity. Probody technology encompasses multiple protease-activatable antibody formats, including monoclonal antibodies, ADCs and BiTEs. In contrast, Conditional Bispecific Redirected Activation (COBRA) refers to a specific conditionally activated TCE platform. Thus, both approaches can incorporate tumor-associated protease-dependent activation mechanisms. In a recent preclinical study, Boustany et al. [86] developed a bispecific CD3-EGFR TCE and studied its profile in vitro and in vivo against colon cancer, confirming its strong antitumor activity with very few side effects. Another conditionally activated EGFR×CD3 TCE, TAK-186, demonstrated significant tumor regression in preclinical models of EGFR-positive solid tumors [87]. TAK-186 subsequently entered phase I/II clinical evaluation (NCT04844073), including an HNSCC expansion cohort; however, the study was terminated following a sponsor decision based on limited anti-cancer activity. CX-904, another conditionally activated EGFR×CD3 TCE, also entered early clinical development (NCT05387265), but its phase I study was subsequently terminated following a sponsor decision. This approach is designed to permit greater systemic exposure while reducing, rather than eliminating, off-tumor immune activation; whether this translates into a consistently improved therapeutic index requires clinical validation.

Switchable TCEs are an advanced form of bispecific antibodies designed to provide dynamic and regulated activation of T cells against tumors. Unlike conventional TCEs, which continuously engage T cells once administered, switchable TCEs incorporate a molecular “on/off” system that allows external regulation of their activity. This is typically achieved through a two-component design, one that binds tumor antigen, and the other that engages CD3 on T cells, but these components only become functionally active when a specific switch molecule, such as a small molecule dimerizer or adaptor, is administered. This modular approach enables precise temporal and spatial control of T cells activation, which can reduce the risk of CRS and off-tumor toxicity. As an example, in a preclinical study a supramolecular HER2×CD3 TCE could be reversibly disassembled by amantadine, providing pharmacologic control over T cells engagement and reducing off-tumor toxicity while preserving antitumor activity [88]. In this context, conditionally activatable and switchable TCEs exemplify how molecular engineering can transform continuous, system-wide T cell activation into a tunable and reversible process tailored to the heterogeneous and anatomically constrained nature of solid tumors such as HNSCC. Logic-gated immune-cell redirection aims to improve discrimination between malignant and normal tissues by integrating multiple antigen-recognition or activation signals. However, dual-antigen binding does not, by itself, establish strict Boolean AND-gate behavior. In soluble TCEs, experimentally investigated approaches, including split-CD3 and affinity-tuned dual-antigen designs, can favor productive T cells activation when selected tumor antigens are co-expressed. Such AND-like behavior is dependent on molecular architecture, antigen density, binding affinity, and the activation threshold, and must be experimentally demonstrated for each construct. Importantly, co-expression of the relevant antigens in normal tissues may still permit off-tumor activity. These designs aim to provide contextual control of immune engagement and thereby improve discrimination between malignant and normal tissues. Boolean logic gating is most extensively established in engineered cellular therapies, particularly CAR-T cells. Related principles have been demonstrated in soluble TCEs, including dual-target and synapse-gated constructs, but the evidence remains predominantly preclinical and substantially less mature than that available for engineered cell therapies [58].

## 9. Synergy with ICB and TME Modulation

ICEs do not act in isolation; they interface dynamically with the checkpoint and TME ecosystems. Checkpoint blockade, via PD-1, PD-L1, or CTLA-4 inhibition, has validated the immunologic relevance of HNSCC, but response rates remain modest and confined to a subset of patients [17,18,89,90]. ICEs may complement checkpoint blockade by providing direct immune-cell redirection and by sustaining local effector-cell activation within the TME [41,91].

TCEs and NKCEs induce immunogenic tumor cell death, releasing neoantigens and danger-associated molecular patterns that prime DCs [92,93]. When combined with PD-1 blockade, this process can overcome exhaustion of both redirected and endogenous T cells. Preclinical studies show that the addition of anti-PD-1 antibodies to bispecific TCEs amplifies cytotoxicity and sustains effector proliferation, especially in PD-L1-positive or INF-suppressed tumors [41]. Crucially, this bidirectional synergy may also reshape the spatial organization of immune infiltrates, facilitating closer effector-tumor cell proximity and more efficient immunologic synapse formation within the HNSCC’s TME. An emerging complementary strategy is to directly manipulate immune-cell spatial interactions. For example, a bispecific dendritic-cell–T-cell engager designed to promote physical interactions between PD-1^+^ T cells and cDC1 enhanced intratumoral immune crosstalk and antitumor immunity in preclinical models [94].

Beyond ICIs, oncolytic virotherapy has emerged as a potent TME modulator. Engineered vaccinia or adenoviral vectors can lyse tumor cells, secrete immune-stimulatory cytokines, and remodel suppressive myeloid compartments [95,96]. When armed with ICE payloads, such viruses serve as biologic delivery vehicles, confining potent engagers to tumor sites while simultaneously inflaming the stroma [64,97].

Similarly, radiotherapy acts as an immune primer by enhancing antigen release, upregulating MHC molecules, and activating the cyclic GMP-AMP synthase-stimulator of interferon genes (cGAS–STING) signaling [98]. The immunomodulatory effects of radiotherapy provide a rationale for combinations with immune-based therapies, including checkpoint blockade; whether these effects can be effectively exploited together with ICEs in HNSCC is to be defined [99].

## 10. Biomarkers for Patient Selection and Pharmacodynamic Monitoring

Biomarker development for ICEs in HNSCC remains largely exploratory. Currently, PD-L1 expression is the only clinically established biomarker used to guide checkpoint-based immunotherapy, whereas the others discussed below have primarily been investigated in the context of ICI therapy or HNSCC prognosis, and their potential role in ICE-based treatment requires prospective validation:

1. PD-L1 expression remains the most established indicator of checkpoint sensitivity, yet its predictive power improves substantially when spatial context and immune-cell composition are incorporated [100,101,102].
2. Gene-expression signatures, such as INF-γ models, and molecular subtype stratifications (Cl6) have shown associations with outcome and response to ICI [103].
3. APOBEC mutational signatures, common in HPV-negative tumors, are associated with increased inflammation and PD-L1 expression and could represent a potential marker of an immunologically distinct tumor subset [16,104].
4. TLSs have emerged as promising spatial biomarkers in HNSCC. Higher TLS abundance and specific spatial features have been associated with favorable outcome and response to ICI. Preliminary studies suggest that TLS-related metrics may provide information complementary to PD-L1 CPS. In ICE trials, serial assessment of TLS composition and organization could represent an exploratory pharmacodynamic endpoint rather than an established marker of response [13,105,106,107].
5. Spatially resolved metrics can quantify the organization and proximity of tumor- and immune-cell populations and have been investigated as biomarkers of immune contexture and response to immunotherapy in HNSCC [13,14,108].
6. Circulating biomarkers provide a minimally invasive means of longitudinal monitoring. In HPV-associated OPSCC, rapid clearance of circulating tumor HPV DNA during treatment has been associated with improved disease control, while tumor-informed and plasma-based ctDNA profiling is increasingly feasible in both viral and non-viral HNSCC. In parallel, molecular characterization of circulating tumor cells has shown potential as a dynamic prognostic and pharmacodynamic biomarker [109,110,111,112,113].
7. Single-cell and TCR-clonotype analyses can identify tumor-resident T cell populations associated with response to immunotherapy. In HNSCC, pre-existing tumor-resident T cells with cytotoxic potential have been associated with response to neoadjuvant PD-1 blockade [114].

Importantly, no single biomarker is likely to be sufficient in isolation; rather, the convergence of spatial, genomic, and dynamic readouts may be required to accurately capture immune engagement and predict durable benefit from ICE-based therapies in HNSCC. Such integrated biomarker frameworks will be critical for adaptive trial designs, enabling dynamic cohort enrichment and early go/no-go decisions based on molecular response rather than radiographic delay.

## 11. The Clinical Landscape: Checkpoint Blockade as the Foundation for ICE Integration

The integration of ICEs with ICB aims to amplify effector function at the tumor-immune interface. In this setting, ICE-based combinations may function less as independent cytotoxic agents and more as amplifiers of pre-existing antitumor immunity, extending the depth and durability of responses initiated by ICB.

HPV-positive tumors, rich in PD-1^+^ CD8^+^ TILs and PD-L1^+^ macrophages, display adaptive immune resistance, providing fertile ground for combinatorial strategies that couple checkpoint relief with T cell or NK cell redirection. Early-phase trials are now prioritizing these inflamed subsets for proof-of-mechanism studies [115,116,117].

Overall, the current clinical landscape highlights both the promise and the challenges of ICE development in solid tumors. Although most platforms have demonstrated manageable safety profiles and proof-of-mechanism, ORR have generally remained poor, and few agents have progressed beyond early-phase clinical development. Importantly, several programs have been discontinued or deprioritized, reflecting not only biological and clinical challenges, but also a matter of drug development, where commercial priorities and portfolio optimization frequently influence program continuation. Collectively, these studies suggest that future progress will likely depend less on further increasing engager potency and more on improving tumor selectivity, TME modulation, and biomarker-guided patient selection.

The clinical experience accumulated to date provides an important reality check for ICE development in HNSCC. Despite compelling preclinical activity, translation into a favorable therapeutic index has been complicated. TAK-186 entered phase I/II evaluation but was subsequently terminated [NCT04844073], while further development of the EGFR×CD3 TCE JANX008 [NCT05783622] was discontinued following early clinical evaluation. Importantly, trial termination and sponsor decisions to discontinue development should be distinguished, and neither should automatically be interpreted as definitive evidence of clinical inefficacy in HNSCC. These experiences underscore that conditional activation alone may be insufficient to overcome limited intratumoral activity, heterogeneous antigen expression, immune exclusion, and other mechanisms of resistance within the TME.

Conversely a 5T4×CD3 TCE (GEN1044) illustrates a different limitation of immune-cell redirection: development was discontinued after the maximum tolerated dose was reached below the exposure expected to provide adequate clinical activity [NCT04424641]. This suggests that limitations in the therapeutic window may prevent potentially effective drug exposure. However, HNSCC-specific efficacy outcomes were not separately reported, precluding conclusions regarding its activity in this tumor population.

At the same time, ongoing programs indicate that the field is continuing to diversify beyond conventional surface-antigen TCEs. CRPA1A2 [NCT07583316] and CLSP-1025 [NCT06778863] are designed to recognize peptide–HLA complexes, potentially enabling the targeting of intracellular tumor antigens in appropriately selected patients. However, eligibility for these trials should not be equated with documented enrollment of patients with HNSCC, and HNSCC-specific clinical outcomes have not yet been reported. Other approaches, including conditionally activated immunotherapies and TME-directed cytokine platforms such as XTX301, are also being investigated, although their clinical benefit in HNSCC remains unestablished [118,119]. Whether these strategies can produce a clinically meaningful separation between efficacy and toxicity remains an open question.

Table 2 summarizes clinical trials of ICEs and conditionally activated drugs in advanced solid tumors, distinguishing trials with documented HNSCC enrollment or outcomes from those in which HNSCC was only an eligible or planned expansion population. Where available, HNSCC-specific efficacy and safety data are reported separately from results obtained in the overall study population.

## 12. Delivery Innovations and TME Conditioning

Beyond molecular design, the route and context of delivery critically shape ICE efficacy.

- Oncolytic virotherapy offers a sophisticated vehicle for in situ immune activation. Enveloped vaccinia platforms exhibit tumor tropism, resist complement inactivation, and remodel vasculature and stroma, facilitating immune-cell entry. When armed with ICE payloads or cytokine cassettes, these vectors effectively transform tumors into localized immune reactors [64]. Radiotherapy may provide a mechanistic rationale for combination with ICEs by promoting tumor-cell death, antigen release, dendritic-cell activation, and changes in inflammatory signaling and immune-cell trafficking. These effects could potentially facilitate immune cells recruitment and ICE-mediated antitumor activity. However, such interactions are context-dependent, and preclinical observations should not be interpreted as proof of concept of synergy between radiotherapy and ICEs. Therefore, the efficacy and safety of concurrent or sequential radiotherapy–ICE combinations in HNSCCs require further validation [99].
- Myeloid reprogramming, through colony stimulating factor 1 receptor (CSF-1R) inhibitors, CD40 agonists, or DC-targeted engagers, can dismantle suppressive macrophage networks, enhance cross-presentation, and extend ICE-induced immunity systemically [94].

Collectively, these delivery and conditioning strategies highlight that effective ICE therapy in HNSCC requires not only target engagement, but deliberate remodeling of vascular, stromal, and behavioral determinants that govern immune access to the tumor.

## 13. Conclusions and Future Perspectives

ICEs represent one of the most rapidly evolving fields in cancer immunotherapy and offer a promising strategy to overcome several limitations of conventional immune checkpoint inhibition in solid tumors. By redirecting endogenous immune effector cells toward TAAs often independently of MHC presentation, these platforms have the potential to broaden the population of patients who may benefit from immunotherapy, particularly those with immune-excluded or poorly inflamed tumors. The preclinical evidence reviewed in this manuscript highlights the remarkable versatility of next-generation ICE engineering. Beyond conventional TCEs, innovative platforms, such as NKCE, γδTCEs, CITEs, multispecific constructs, and protease-activatable antibodies have been developed to improve immune activation, enhance tumor selectivity, and overcome several mechanisms of immune escape. In particular, masked ICEs and conditionally activated platforms appear to be attractive for HNSCCs because they may reduce on-target off-tumor toxicity while preserving potent antitumor activity within the anatomically delicate head and neck region. Despite these encouraging biological advances, clinical translation has so far been more challenging than initially anticipated. Although several early-phase clinical studies have demonstrated proof-of-mechanism and generally manageable safety profiles, only a limited number of ICE platforms have progressed beyond phase I/II development. These findings emphasize that successful immune redirection in solid tumors depends on far more than target recognition alone. Antigen heterogeneity, insufficient immune-cell infiltration, stromal exclusion, T-cell exhaustion, immunosuppressive myeloid populations, and the narrow therapeutic window imposed by physiological antigen expression continue to represent major barriers limiting durable clinical efficacy. The experience gained from early clinical development also suggests that increasing the potency of ICEs is unlikely to be sufficient. Instead, the future of ICE-based immunotherapy will probably rely on rational engineering strategies capable of improving both efficacy and safety. Among these, conditional activation through masking technologies, multispecific or logic-gated constructs, local cytokine delivery, and combination approaches aimed at remodeling the TME seem to be particularly promising. Likewise, biomarker-driven patient selection, incorporating spatial immune organization, antigen expression patterns, and dynamic molecular biomarkers, could become essential for identifying patients most likely to benefit from these engineered therapies. Importantly, HNSCC represents both an attractive opportunity and a challenging disease model for the development of ICEs. The frequent expression of therapeutically relevant antigens, the accessibility of tumor tissue for serial biopsies, and the possibility of locoregional treatment strategies promote translational research. Conversely, profound immune suppression, marked intratumoral heterogeneity, and the close anatomical proximity of tumors to critical structures and organs require therapeutic platforms with exceptional precision and favorable safety profiles. In summary, the available evidence indicates that ICEs have not yet fulfilled their initial promise in HNSCC, but neither have they reached their full clinical potential. Current data support continued clinical development while highlighting the need for more sophisticated engineering, improved patient selection, and rational combination strategies. Future progress will likely depend not only on identifying optimal tumor antigens, but also on integrating tumor-selective immune activation with effective modulation of the TME. Such an approach may ultimately enable ICEs to become an important component of precision immunotherapy for patients with HNSCC.

## Figures and Tables

**Table 1:** Next-generation engineering platforms for immune cell engagers: design principles, mechanisms and rationale for HNSCCs.

Platform	Molecular Construct	Immune Target(s)	Tumor Target(s)	Mechanism of Action	Main Advantages	Development Stage *	Rationale for HNSCC
BiTEs	Bispecific scFv antibody	CD3	Single TAA	Redirects endogenous T cells toward surface tumor antigens independently of peptide-MHC recognition	Potent cytotoxicity; clinically validated platform	Clinical	MHC-independent T-cell redirection may be relevant in HNSCC with impaired antigen presentation; however, activity in solid tumors is limited by T-cell dysfunction, tumor penetration, antigen heterogeneity and toxicity.
Half-life Extended (HLE)-TCEs	Fc-containing or albumin-binding bispecific antibody	CD3	Single TAA	Prolonged T-cell redirection through improved pharmacokinetics	Longer half-life and intermittent dosing	Clinical	Extended systemic exposure and intermittent dosing may improve pharmacologic feasibility compared with short-half-life BiTE formats, although prolonged exposure may also increase the importance of controlling systemic immune activation.
SMITEs	Dual bispecific antibodies	CD3^+^ CD28/4-1BB/OX40	Single or dual TAAs	Simultaneous delivery of activation (Signal 1) and co-stimulation (Signal 2), AND-gate behavior clinically unproven	Enhanced T-cell activation and persistence	Preclinical	Addition of co-stimulatory signaling may enhance T-cell activation in an immunosuppressive HNSCC TME, but clinical benefit has not yet been established.
TITAN	Multispecific nanobody-based engager	CD8^+^ CD3/TCR	Single TAA	Selective recruitment of cytotoxic CD8^+^ T cells while minimizing CD4^+^ activation	Designed to limit CD4-associated cytokine production	Preclinical platform validation; early clinical development (NCT06542250)	Preferential recruitment of CD8^+^ T cells may reduce CD4-associated cytokine production and potentially improve therapeutic index, a relevant consideration in anatomically sensitive HNSCC.
CITEs	Bispecific/trispecific antibody with checkpoint blockade	CD3^+^ PD-1/PD-L1 (or CTLA-4)	Single TAA	Simultaneous T-cell redirection and local checkpoint inhibition	Restores T-cell activity within immunosuppressive TME	Preclinical/Early clinical	Local integration of T-cell redirection and checkpoint inhibition may be attractive in PD-L1-expressing HNSCC, but superiority over separate TCE plus ICI administration remains unproven.
TriKEs	Trispecific NK cell-engager incorporating IL-15	CD16^+^ IL-15	Single TAA	Redirects NK cells while promoting their proliferation and persistence	Enhanced NK-cell activation, proliferation and survival	Clinical	IL-15-containing NK engagers may enhance NK-cell proliferation and function; this is relevant because NK dysfunction occurs in HNSCC, although restoration of durable NK activity in the suppressive TME remains unproven.
BiKEs	Tetravalent bispecific NK-cell engagers	CD16A	Single TAA	Potent NK-cell-mediated ADCC	High avidity and efficient NK-cell activation	Clinical	MHC-independent NK-cell redirection may complement T-cell-based approaches, particularly in tumors with antigen-presentation defects; HNSCC-specific clinical evidence remains limited.
γδTCEs	Bispecific antibody	Vγ9Vδ2 TCR or BTN2A1/BTN3A1	Single TAA	Redirects γδT cells independently of classical MHC presentation	Activity against MHC-deficient tumors	Preclinical	MHC-independent γδT cell recognition provides a rationale in HNSCC with antigen-presentation defects, but efficacy and durability in the HNSCC TME remain clinically unvalidated.
Protease-activatable (Masked) TCEs/COBRA	Conditionally activated bispecific antibody	CD3	Single TAA	Tumor-associated proteases cleave a masking peptide, activating the engager preferentially within the TME	Reduce systemic exposure to off-tumor toxicity	Early clinical	Conditional activation may widen the therapeutic index of highly potent TCEs, particularly for epithelial targets such as EGFR that are also expressed in normal tissues. This is especially relevant in the anatomically constrained head-and-neck region.
Probody^®^	Protease-cleavable masked antibody (may include monoclonal antibodies, ADCs or bispecific engagers)	Variable	Variable	Preferential activation following proteolytic mask cleavage	Improved therapeutic index remains platform-dependent; potential to reduce systemic target engagement	Clinical	Protease-dependent activation offers a strategy to reduce systemic target engagement; however, clinical experience has shown that improved selectivity does not necessarily translate into sufficient antitumor activity.
Switchable TCEs	Modular adaptor-controlled bispecific antibody	CD3	Single TAA	Immune activation is externally controlled by a small-molecule switch	Temporal control of T-cell activation	Preclinical	Reversible pharmacologic control could allow rapid interruption of immune engagement if toxicity develops, which is conceptually attractive where local inflammation may compromise critical structures.
Logic-gated ICEs (AND/OR/NOT)	Multispecific engineered antibodies	CD3 ± co-stimulatory receptors	Multiple TAAs	Activation requires predefined antigen combinations	Potential for improved discrimination between antigen expression patterns; reduced off-tumor toxicity not clinically established	Preclinical	Multisignal recognition may improve discrimination between tumor and normal tissues and address antigen heterogeneity, both important limitations in HNSCC.
Armored CAR-secreting BiTEs	CAR-T cells engineered to secrete BiTEs	Endogenous T cells + CAR-T cells	Single TAA	Local release of BiTEs recruits bystander T cells and amplifies immune responses	Combines adoptive cell therapy with endogenous immune recruitment	Preclinical/Early clinical	Local production of engagers may increase intratumoral immune recruitment while limiting systemic exposure, although manufacturing complexity and trafficking remain major barriers.

Legend: CD = cluster differentiation; HNSCC = head and neck squamous cell carcinoma; TAA = tumor associated antigen; IL = interleukin; PD = programmed death; PD-L1 = programmed death-ligand 1; CTLA-4 = cytotoxic T-lymphocyte antigen 4; ADCC = antibody-dependent cellular cytotoxicity; TCR = T-cell receptor; ICE = immune-cell engager; TCE = T-cell engager; BiTE = bispecific T-cell engager; γδTCE = gamma delta T-cell engager; CITEs = Checkpoint-Inhibitory T-cell engagers; SMITEs = Simultaneous Multiple Interaction T-cell Engagers; TITAN = Targeting Induced T-cell Activating Nanobodies; COBRA = conditional bispecific redirected activation; CAR = chimeric antigen receptor; BTN = butyrophilin. * Development stage indicates the most advanced stage reached by the platform across oncology and does not necessarily reflect clinical development in HNSCC. HNSCC-specific maturity is described separately in the ‘Rationale for HNSCC’ column.

**Table 2:** Clinical trials of ICEs and conditionally activated therapeutics in advanced solid tumors, with potential eligibility of HNSCCs or documented results for HNSCCs cohorts.

Registry ID(s)	Agent/Platform	Mechanism/Targets	Phase	Combination	Results
***ICE DRUGS***					
NCT07583316	CRPA1A2	MAGE-A1/HLA-A*02:01 BiTE	I	Monotherapy	Registry status: not yet recruiting (update May 2026). HNSCC is eligible in the biomarker-selected solid-tumor population; no treated HNSCC cohort or clinical outcomes reported (HNSCC eligibility does not establish enrollment).
NCT05783622	JANX008	Conditionally activated EGFR×CD3 TCE	I	Monotherapy	Registry status: active, not recruiting (update May 2026, accessed October 2026). Further clinical development discontinued by sponsor following Phase 1a evaluation. Registry trial status should be distinguished from program discontinuation. HNSCC was an eligible tumor type, HNSCC-specific efficacy results have not been separately reported.
NCT04844073	TAK-186	EGFR×CD3 TCE	I/II	± anti-PD-1	Registry status: terminated due to limited anticancer activity (95 participants enrolled). Registered results posted September 2026: no confirmed CR or PR across reported dose groups (overall-study results, not HNSCC-specific). Program: discontinued following limited monotherapy antitumor activity (sponsor decision). HNSCC was an eligible tumor type, but HNSCC-specific efficacy results were not separately reported
NCT04424641	GEN1044	5T4×CD3 bispecific, Fc-silenced IgG1 antibody	I	Monotherapy	Study stopped during dose escalation following safety-committee review of the overall safety profile and emerging safety signals, according to EU trial results; planned expansion was not initiated; subsequent program discontinuation should be distinguished from the trial-level decision. HNSCC was an eligible tumor type, but HNSCC-specific efficacy results were not separately reported
NCT06778863	CLSP-1025	TCE targeting p53 R175H mutation	I	Monotherapy	Registry status: Recruiting (update February 2026). No HNSCC-specific treated population or clinical outcomes publicly reported; eligibility is biomarker- and protocol-dependent.
***MASKED IMMUNOTHERAPEUTIC DRUGS***					
NCT03543813	CX-2029 (Probody-ADC) [120]	Masked anti-CD71 ADC (tumor protease-activated)	I/II	Monotherapy	Overall Phase I dose escalation (mixed solid tumors; 45 patients): four DLTs (2/4 at 5 mg/kg and 2/6 at 4 mg/kg); these are not HNSCC-specific. HNSCC expansion (29 October 2021 cutoff; sponsor-reported): 29 treated/safety-evaluable, 25 efficacy-evaluable; 1 confirmed PR (ORR 4.0%); DCR 56.0% (14/25). Registry status: completed.
NCT03149549	CX-2009 (Praluzatamab Ravtansine) [121]	Masked anti-CD166 Probody ADC	I/II	±ICIs (per protocol version)	Overall study: 99 treated; 80 response-evaluable; 3% DLT, 33% serious adverse events; 10 PR, 10 SD. HNSCC expansion cohort (9 pts): one unconfirmed PR, ORR not established. Registry status: termination related to COVID-19/business restructuring (no Phase II), not reported lack of efficacy
NCT05684965	XTX301	Masked, tumor-activated IL-12 (MMP-cleavable)	I/II	Monotherapy	Registry status: Recruiting (update June 2026); broader development-program status requires separate verification (no discontinuation reason is inferred). HNSCC was an eligible/planned expansion population. No separately documented HNSCC enrollment, efficacy or safety results in the reviewed sources.

Legend: ADC = antibody-drug conjugated; DLT = dose limiting toxicity; CR = complete response; PR = partial response; SD = stable disease; PD = progression disease; ORR = objective response rate; DCR = disease control rate. Notes: The absence of publicly available results for several studies should not be interpreted as evidence of treatment failure but rather reflects the early stage of development of many ICE platforms and the delay between trial completion and public disclosure of efficacy and safety data. HNSCC-specific enrollment and efficacy results are reported only when explicitly documented in published studies, conference abstracts, clinical-trial records, or sponsor-reported datasets. Eligibility of patients with HNSCC does not establish actual enrollment. Safety findings refer to the overall study population unless explicitly identified as HNSCC-specific. Denominators correspond to the population evaluable for each endpoint and may differ between safety and efficacy analyses. Data cutoffs and evidence sources are specified where relevant.

## Data Availability

No new data were created or analyzed in this study. Data sharing is not applicable to this article.

## References

[1] Meng X., Zheng Y., Zhang L., Liu P., Liu Z., He Y. (2024). Single-Cell Analyses Reveal the Metabolic Heterogeneity and Plasticity of the Tumor Microenvironment during Head and Neck Squamous Cell Carcinoma Progression. Cancer Res..

[2] Gong X., Xiong J., Gong Y., Zhang J., Zhang J., Yang G., Chi H., Tian G. (2023). Deciphering the role of HPV-mediated metabolic regulation in shaping the tumor microenvironment and its implications for immunotherapy in HNSCC. Front. Immunol..

[3] Jin S., Li R., Chen M.Y., Yu C., Tang L.Q., Liu Y.M., Li J.P., Liu Y.N., Luo Y.L., Zhao Y. (2020). Single-cell transcriptomic analysis defines the interplay between tumor cells, viral infection, and the microenvironment in nasopharyngeal carcinoma. Cell Res..

[4] de la Iglesia J.V., Slebos R.J.C., Martin-Gomez L., Wang X., Teer J.K., Tan A.C., Gerke T.A., Aden-Buie G., van Veen T., Masannat J. (2020). Effects of Tobacco Smoking on the Tumor Immune Microenvironment in Head and Neck Squamous Cell Carcinoma. Clin. Cancer Res..

[5] Chung C.H., de la Iglesia J.V., Wang X., Song F., Chaudhary R., Masannat J., Conejo-Garcia J., Hernandez-Prera J., Slebos R. (2019). Tobacco smoking is associated with the immune suppressive microenvironment in head and neck squamous cell carcinoma (HNSCC). Ann. Oncol..

[6] Jing F., Wang J., Zhou L., Ning Y., Xu S., Zhu Y. (2021). Bioinformatics analysis of the role of CXC ligands in the microenvironment of head and neck tumor. Aging.

[7] Gu J.T., Claudio N., Betts C., Sivagnanam S., Geltzeiler M., Pucci F. (2022). Characterization of the tumor immune microenvironment of sinonasal squamous-cell carcinoma. Int. Forum Allergy Rhinol..

[8] Barsouk A., Aluru J.S., Rawla P., Saginala K., Barsouk A. (2023). Epidemiology, Risk Factors, and Prevention of Head and Neck Squamous Cell Carcinoma. Med. Sci..

[9] Bhatia A., Burtness B. (2023). Treating Head and Neck Cancer in the Age of Immunotherapy: A 2023 Update. Drugs.

[10] Ayers M., Lunceford J., Nebozhyn M., Murphy E., Loboda A., Kaufman D.R., Albright A., Cheng J.D., Kang S.P., Shankaran V. (2017). IFN-γ-related mRNA profile predicts clinical response to PD-1 blockade. J. Clin. Investig..

[11] Johnson D.E., Burtness B., Leemans C.R., Lui V.W.Y., Bauman J.E., Grandis J.R. (2020). Head and neck squamous cell carcinoma. Nat. Rev. Dis. Primers.

[12] Offner S., Hofmeister R., Romaniuk A., Kufer P., Baeuerle P.A. (2006). Induction of regular cytolytic T cell synapses by bispecific single-chain antibody constructs on MHC class I-negative tumor cells. Mol. Immunol..

[13] Sadeghirad H., Liu N., Monkman J., Ma N., Ben Cheikh B., Jhaveri N., Tan C.W., Warkiani M.E., Adams M.N., Nguyen Q. (2023). Compartmentalized spatial profiling of the tumor microenvironment in head and neck squamous cell carcinoma identifies immune checkpoint molecules and tumor necrosis factor receptor superfamily members as biomarkers of response to immunotherapy. Front. Immunol..

[14] Henry L.E., Krishnan S., Patil S., Bellile E., Perera C.D., Li S., Qin T., Taylor J.M.G., Rao A., Chinn S.B. (2023). Spatial relationships and immune subsets in the tumor immune microenvironment of head and neck cancers. J. Clin. Oncol..

[15] Liu Z., Meng X., Tang X., Zou W., He Y. (2023). Intratumoral tertiary lymphoid structures promote patient survival and immunotherapy response in head neck squamous cell carcinoma. Cancer Immunol. Immunother. CII.

[16] Messerschmidt C., Obermayer B., Klinghammer K., Ochsenreither S., Treue D., Stenzinger A., Glimm H., Fröhling S., Kindler T., Brandts C.H. (2020). Distinct immune evasion in APOBEC-enriched, HPV-negative HNSCC. Int. J. Cancer.

[17] Burtness B., Harrington K.J., Greil R., Soulières D., Tahara M., de Castro G., Psyrri A., Basté N., Neupane P., Bratland Å. (2019). Pembrolizumab alone or with chemotherapy versus cetuximab with chemotherapy for recurrent or metastatic squamous cell carcinoma of the head and neck (KEYNOTE-048): A randomised, open-label, phase 3 study. Lancet.

[18] Ferris R.L., Blumenschein G., Fayette J., Guigay J., Colevas A.D., Licitra L., Harrington K., Kasper S., Vokes E.E., Even C. (2016). Nivolumab for Recurrent Squamous-Cell Carcinoma of the Head and Neck. N. Engl. J. Med..

[19] Ribas A., Wolchok J.D. (2018). Cancer immunotherapy using checkpoint blockade. Science.

[20] Binnewies M., Roberts E.W., Kersten K., Chan V., Fearon D.F., Merad M., Coussens L.M., Gabrilovich D.I., Ostrand-Rosenberg S., Hedrick C.C. (2018). Understanding the tumor immune microenvironment (TIME) for effective therapy. Nat. Med..

[21] Labrijn A.F., Janmaat M.L., Reichert J.M., Parren P.W.H.I. (2019). Bispecific antibodies: A mechanistic review of the pipeline. Nat. Rev. Drug Discov..

[22] Goebeler M.E., Bargou R.C. (2020). T cell-engaging therapies—BiTEs and beyond. Nat. Rev. Clin. Oncol..

[23] Mack M., Riethmüller G., Kufer P. (1995). A small bispecific antibody construct expressed as a functional single-chain molecule with high tumor cell cytotoxicity. Proc. Natl. Acad. Sci. USA.

[24] Garrido F., Aptsiauri N., Doorduijn E.M., Garcia Lora A.M., van Hall T. (2016). The urgent need to recover MHC class I in cancers for effective immunotherapy. Curr. Opin. Immunol..

[25] Bargou R., Leo E., Zugmaier G., Klinger M., Goebeler M., Knop S., Noppeney R., Viardot A., Hess G., Schuler M. (2008). Tumor regression in cancer patients by very low doses of a T cell-engaging antibody. Science.

[26] Kantarjian H., Stein A., Gökbuget N., Fielding A.K., Schuh A.C., Ribera J.M., Wei A., Dombret H., Foà R., Bassan R. (2017). Blinatumomab versus Chemotherapy for Advanced Acute Lymphoblastic Leukemia. N. Engl. J. Med..

[27] Gauthier L., Morel A., Anceriz N., Rossi B., Blanchard-Alvarez A., Grondin G., Trichard S., Cesari C., Sapet M., Bosco F. (2019). Multifunctional Natural Killer Cell Engagers Targeting NKp46 Trigger Protective Tumor Immunity. Cell.

[28] Vivier E., Raulet D.H., Moretta A., Caligiuri M.A., Zitvogel L., Lanier L.L., Yokoyama W.M., Ugolini S. (2011). Innate or adaptive immunity? The example of natural killer cells. Science.

[29] Mele D., Pessino G., Trisolini G., Luchena A., Benazzo M., Morbini P., Mantovani S., Oliviero B., Mondelli M.U., Varchetta S. (2022). Impaired intratumoral natural killer cell function in head and neck carcinoma. Front. Immunol..

[30] Silva-Santos B., Mensurado S., Coffelt S.B. (2019). γδ T cells: Pleiotropic immune effectors with therapeutic potential in cancer. Nat. Rev. Cancer.

[31] Rigau M., Ostrouska S., Fulford T.S., Johnson D.N., Woods K., Ruan Z., McWilliam H.E.G., Hudson C., Tutuka C., Wheatley A.K. (2020). Butyrophilin 2A1 is essential for phosphoantigen reactivity by γδ T cells. Science.

[32] van Diest E., Hernández López P., Meringa A.D., Vyborova A., Karaiskaki F., Heijhuurs S., Gumathi Bormin J., van Dooremalen S., Nicolasen M.J.T., Gatti L.C.D.E. (2021). Gamma delta TCR anti-CD3 bispecific molecules (GABs) as novel immunotherapeutic compounds. J. Immunother. Cancer.

[33] Weiskopf K., Weissman I.L. (2015). Macrophages are critical effectors of antibody therapies for cancer. mAbs.

[34] Chen D.S., Mellman I. (2013). Oncology meets immunology: The cancer-immunity cycle. Immunity.

[35] Mpekris F., Voutouri C., Baish J.W., Duda D.G., Munn L.L., Stylianopoulos T., Jain R.K. (2020). Combining microenvironment normalization strategies to improve cancer immunotherapy. Proc. Natl. Acad. Sci. USA.

[36] Hegde P.S., Chen D.S. (2020). Top 10 Challenges in Cancer Immunotherapy. Immunity.

[37] Cramer J.D., Burtness B., Ferris R.L. (2019). Immunotherapy for head and neck cancer: Recent advances and future directions. Oral Oncol..

[38] Deegen P., Thomas O., Nolan-Stevaux O., Li S., Wahl J., Bogner P., Aeffner F., Friedrich M., Liao M.Z., Matthes K. (2021). The PSMA-targeting Half-life Extended BiTE Therapy AMG 160 has Potent Antitumor Activity in Preclinical Models of Metastatic Castration-resistant Prostate Cancer. Clin. Cancer Res..

[39] Brinkmann U., Kontermann R.E. (2017). The making of bispecific antibodies. mAbs.

[40] Spiess C., Zhai Q., Carter P.J. (2015). Alternative molecular formats and therapeutic applications for bispecific antibodies. Mol. Immunol..

[41] Tapia-Galisteo A., Sánchez-Rodríguez I., Narbona J., Iglesias-Hernández P., Aragón-García S., Jiménez-Reinoso A., Compte M., Khan S., Tsuda T., Chames P. (2024). Combination of T cell-redirecting strategies with a bispecific antibody blocking TGF-β and PD-L1 enhances antitumor responses. Oncoimmunology.

[42] Enderle L., Shalaby K.H., Gorelik M., Weiss A., Blazer L.L., Paduch M., Cardarelli L., Kossiakoff A., Adams J.J., Sidhu S.S. (2021). A T cell redirection platform for co-targeting dual antigens on solid tumors. mAbs.

[43] Zhao P., Schardt J., Chiang C.I., Shah P., Eun G.S., Martinek J., Cyr M., Johnson Y., Amofah B., Ye X. (2025). Improving dual targeting selectivity in T-cell engagers via synapse-gated and affinity-tuned trispecific antibody design. mAbs.

[44] Mason D.M., Friedensohn S., Weber C.R., Jordi C., Wagner B., Meng S.M., Ehling R.A., Bonati L., Dahinden J., Gainza P. (2021). Optimization of therapeutic antibodies by predicting antigen specificity from antibody sequence via deep learning. Nat. Biomed. Eng..

[45] Roybal K.T., Rupp L.J., Morsut L., Walker W.J., McNally K.A., Park J.S., Lim W.A. (2016). Precision Tumor Recognition by T Cells with Combinatorial Antigen-Sensing Circuits. Cell.

[46] Williams J.Z., Allen G.M., Shah D., Sterin I.S., Kim K.H., Garcia V.P., Shavey G.E., Yu W., Puig-Saus C., Tsoi J. (2020). Precise T cell recognition programs designed by transcriptionally linking multiple receptors. Science.

[47] Li J., Piskol R., Ybarra R., Chen Y.J.J., Li J., Slaga D., Hristopoulos M., Clark R., Modrusan Z., Totpal K. (2019). CD3 bispecific antibody—Induced cytokine release is dispensable for cytotoxic T cell activity. Sci. Transl. Med..

[48] Lee D.W., Santomasso B.D., Locke F.L., Ghobadi A., Turtle C.J., Brudno J.N., Maus M.V., Park J.H., Mead E., Pavletic S. (2019). ASTCT Consensus Grading for Cytokine Release Syndrome and Neurologic Toxicity Associated with Immune Effector Cells. Biol. Blood Marrow Transplant..

[49] Brudno J.N., Kochenderfer J.N. (2016). Toxicities of chimeric antigen receptor T cells: Recognition and management. Blood.

[50] Laura Q.M., Chow M.D. (2020). Head and Neck Cancer. N. Engl. J. Med..

[51] Morris E.C., Neelapu S.S., Giavridis T., Sadelain M. (2022). Cytokine release syndrome and associated neurotoxicity in cancer immunotherapy. Nat. Rev. Immunol..

[52] Cattaruzza F., Nazeer A., To M., Hammond M., Koski C., Liu L.Y., Pete Yeung V., Rennerfeldt D.A., Henkensiefken A., Fox M. (2023). Precision-activated T-cell engagers targeting HER2 or EGFR and CD3 mitigate on-target, off-tumor toxicity for immunotherapy in solid tumors. Nat. Cancer.

[53] Nathan P., Hassel J.C., Rutkowski P., Baurain J.F., Butler M.O., Schlaak M., Sullivan R.J., Ochsenreither S., Dummer R., Kirkwood J.M. (2021). Overall Survival Benefit with Tebentafusp in Metastatic Uveal Melanoma. N. Engl. J. Med..

[54] Minocha M., Thompson C.G., Murphy A., Zhou Y., Brandl C., Parkes A., Chen X., Yu B., Martinez P., Houk B.E. (2024). Pharmacokinetics of Tarlatamab, a Delta-Like Ligand-3 (DLL3) Targeted Half-Life Extended Bispecific T-Cell Engager (BiTE^®^) Immunotherapy in Adult Patients with Previously Treated Small-Cell Lung Cancer: Results from DeLLphi-300, a Phase I Multiple-Dose-Escalation Study. Clin. Pharmacokinet..

[55] Ahn M.J., Cho B.C., Felip E., Korantzis I., Ohashi K., Majem M., Juan-Vidal O., Handzhiev S., Izumi H., Lee J.S. (2023). Tarlatamab for Patients with Previously Treated Small-Cell Lung Cancer. N. Engl. J. Med..

[56] Tapia-Galisteo A., Sánchez Rodríguez Í., Aguilar-Sopeña O., Harwood S.L., Narbona J., Ferreras Gutierrez M., Navarro R., Martín-García L., Corbacho C., Compte M. (2022). Trispecific T-cell engagers for dual tumor-targeting of colorectal cancer. Oncoimmunology.

[57] Correnti C.E., Laszlo G.S., de van der Schueren W.J., Godwin C.D., Bandaranayake A., Busch M.A., Gudgeon C.J., Bates O.M., Olson J.M., Mehlin C. (2018). Simultaneous multiple interaction T-cell engaging (SMITE) bispecific antibodies overcome bispecific T-cell engager (BiTE) resistance via CD28 co-stimulation. Leukemia.

[58] Shen Y., Jin S.J., Chen Y.C., Liu W.H., Li Y.M., Zhao W.Y., Xu Y.C., Chen S.Q., Zhao W.B. (2023). Improving the tumor selectivity of T cell engagers by logic-gated dual tumor-targeting. Pharmacol. Res..

[59] Nolan-Stevaux O., Smith R. (2024). Logic-Gated and Contextual Control of Immunotherapy for Solid Tumors: Contrasting Multi-Specific T Cell Engagers and CAR-T Cell Therapies. Front. Immunol..

[60] Cayatte C., Ciucci T., Lin W., Lara A., Jafarzadeh N., Foreman T., Lisiero D., Hamilton T., Campbell D., Jelicic K. (2024). 1093 Development of TITAN: A new CD8-guided T cell engager platform for hematological and solid tumor applications. J. Immunother. Cancer.

[61] Shadman M., Thompson P., Heyman B., Hutchings M., Lori L.A., Yuda J., Zinzani P.L., Colton D., Hoehn D., Izuzquiza M. (2025). TITANium: An Open-Label, Global Multicenter Phase 1/2 Study of AZD5492, a First-in-Class Subcutaneous CD8-Guided Tri-Specific T-Cell Engager (TCE), in Patients (Pts) with Relapsed or Refractory (r/r) B-Cell Malignancies. J. Clin. Oncol..

[62] Szijj P.A., Gray M.A., Ribi M.K., Bahou C., Nogueira J.C.F., Bertozzi C.R., Chudasama V. (2023). Chemical generation of checkpoint inhibitory T cell engagers for the treatment of cancer. Nat. Chem..

[63] Saliu M.A., Wang Q., Salisu M.D., Ren Y., Zhang P., Suleiman R.B., Cao B., Xu Y., Liu X., Lluis F. (2025). Mesothelin-targeted CAR-T cells secreting NKG2D-BiTEs exhibit potent efficacy against triple-negative breast cancer. Exp. Hematol. Oncol..

[64] Zhu H., Zhang W., Guo Q., Fan R., Luo G., Liu Y. (2024). Engineered oncolytic virus expressing B7H3-targeting BiTE enhances antitumor T-cell immune response. J. Immunother. Cancer.

[65] Wingert S., Reusch U., Knackmuss S., Kluge M., Damrat M., Pahl J., Schniegler-Mattox U., Mueller T., Fucek I., Ellwanger K. (2021). Preclinical evaluation of AFM24, a novel CD16A-specific innate immune cell engager targeting EGFR-positive tumors. mAbs.

[66] Khaw M.J., Zorko N.A., Kennedy P.R., Bendzick L.E., Shackelford M., Selleck C., Hinderlie P., Walker J.T., Soignier Y., Lyons R.C. (2025). Novel trispecific killer engager targeting B7-H3 enhances natural killer cell antitumor activity against head and neck cancer. J. Immunother. Cancer.

[67] Vallera D.A., Ferrone S., Kodal B., Hinderlie P., Bendzick L., Ettestad B., Hallstrom C., Zorko N.A., Rao A., Fujioka N. (2020). NK-Cell-Mediated Targeting of Various Solid Tumors Using a B7-H3 Tri-Specific Killer Engager In Vitro and In Vivo. Cancers.

[68] El-Khoueiry A., Saavedra O., Thomas J., Livings C., Garralda E., Hintzen G., Kohlhas L., Vanosmael D., Koch J., Rajkovic E. (2025). First-in-Human Phase I Study of a CD16A Bispecific Innate Cell Engager, AFM24, Targeting EGFR-Expressing Solid Tumors. Clin. Cancer Res..

[69] Hannani D., Ma Y., Yamazaki T., Déchanet-Merville J., Kroemer G., Zitvogel L. (2012). Harnessing γδ T cells in anticancer immunotherapy. Trends Immunol..

[70] King L.A., Toffoli E.C., Veth M., Iglesias-Guimarais V., Slot M.C., Amsen D., van de Ven R., Derks S., Fransen M.F., Tuynman J.B. (2023). A Bispecific γδ T-cell Engager Targeting EGFR Activates a Potent Vγ9Vδ2 T cell—Mediated Immune Response against EGFR-Expressing Tumors. Cancer Immunol. Res..

[71] Aehnlich P., Carnaz Simões A.M., Skadborg S.K., Holmen Olofsson G., Thor Straten P. (2020). Expansion with IL-15 Increases Cytotoxicity of Vγ9Vδ2 T Cells and Is Associated with Higher Levels of Cytotoxic Molecules and T-bet. Front. Immunol..

[72] Yano S., Kondo K., Yamaguchi M., Richmond G., Hutchison M., Wakeling A., Averbuch S., Wadsworth P. (2003). Distribution and Function of EGFR in Human Tissue and the Effect of EGFR Tyrosine Kinase Inhibition. Anticancer. Res..

[73] Ciulean I.S., Fischer J., Quaiser A., Bach C., Abken H., Tretbar U.S., Fricke S., Koehl U., Schmiedel D., Grunwald T. (2023). CD44v6 specific CAR-NK cells for targeted immunotherapy of head and neck squamous cell carcinoma. Front. Immunol..

[74] Haist C., Schulte E., Bartels N., Bister A., Poschinski Z., Ibach T.C., Geipel K., Wiek C., Wagenmann M., Monzel C. (2021). CD44v6-targeted CAR T-cells specifically eliminate CD44 isoform 6 expressing head/neck squamous cell carcinoma cells. Oral. Oncol..

[75] Kawano T., Nakamura Y., Yanoma S., Kubota A., Furukawa M., Miyagi Y., Tsukuda M. (2004). Expression of E-cadherin, and CD44s and CD44v6 and its association with prognosis in head and neck cancer. Auris Nasus Larynx.

[76] Lei X., Ou Z., Yang Z., Zhong J., Zhu Y., Tian J., Wu J., Deng H., Lin X., Peng Y. (2021). A Pan-Histone Deacetylase Inhibitor Enhances the Antitumor Activity of B7-H3—Specific CAR T Cells in Solid Tumors. Clin. Cancer Res..

[77] Yang S., Cao B., Zhou G., Zhu L., Wang L., Zhang L., Kwok H.F., Zhang Z., Zhao Q. (2020). Targeting B7-H3 Immune Checkpoint with Chimeric Antigen Receptor-Engineered Natural Killer Cells Exhibits Potent Cytotoxicity Against Non-Small Cell Lung Cancer. Front. Pharmacol..

[78] Mao L., Fan T.F., Wu L., Yu G.T., Deng W.W., Chen L., Bu L.L., Ma S.R., Liu B., Bian Y. (2017). Selective blockade of B7-H3 enhances antitumour immune activity by reducing immature myeloid cells in head and neck squamous cell carcinoma. J. Cell Mol. Med..

[79] Lin W., Xu Y., Gao J., Zhang H., Sun Y., Qiu X., Huang Q., Kong L., Lu J.J. (2021). Multi-Omics Data Analyses Identify B7-H3 as a Novel Prognostic Biomarker and Predict Response to Immune Checkpoint Blockade in Head and Neck Squamous Cell Carcinoma. Front. Immunol..

[80] Albers A., Abe K., Hunt J., Wang J., Lopez-Albaitero A., Schaefer C., Gooding W., Whiteside T.L., Ferrone S., DeLeo A. (2005). Antitumor activity of human papillomavirus type 16 E7—Specific T cells against virally infected squamous cell carcinoma of the head and neck. Cancer Res..

[81] Dao T., Mun S., Korontsvit T., Khan A.G., Pohl M.A., White T., Klatt M.G., Andrew D., Lorenz I.C., Scheinberg D.A. (2022). A TCR mimic monoclonal antibody for the HPV-16 E7-epitope p11-19/HLA-A*02:01 complex. PLoS ONE.

[82] Wang X., Li Y., Pan M., Lu T., Wang M., Wang Z., Liu C., Hu G. (2022). CEACAM5 inhibits the lymphatic metastasis of head and neck squamous cell carcinoma by regulating epithelial-mesenchymal transition via inhibiting MDM2. Clin. Sci..

[83] Mei Z., Zhang K., Lam A.K.Y., Huang J., Qiu F., Qiao B., Zhang Y. (2020). MUC1 as a target for CAR-T therapy in head and neck squamous cell carinoma. Cancer Med..

[84] Brust L.A., Kühn J.P., Körner S., Knebel M., Braun F.L., Mustapha A.A., Wemmert S., Schick B., Wagner M., Ertz M. (2026). TROP2 expression in head and neck squamous cell carcinoma: Association with the tumor immune microenvironment and clinical outcome. Front. Oncol..

[85] Patel E., Malkova N.V., Crowe D., Pederzoli-Ribeil M., Fantini D., Fanny M., Madala H.R., Jenkins K.A., Yerov O., Greene J. (2024). XTX301, a Tumor-Activated Interleukin-12 Has the Potential to Widen the Therapeutic Index of IL12 Treatment for Solid Tumors as Evidenced by Preclinical Studies. Mol. Cancer Ther..

[86] Boustany L.M., LaPorte S.L., Wong L., White C., Vinod V., Shen J., Yu W., Koditek D., Winter M.B., Moore S.J. (2022). A Probody T Cell—Engaging Bispecific Antibody Targeting EGFR and CD3 Inhibits Colon Cancer Growth with Limited Toxicity. Cancer Res..

[87] Dettling D.E., Kwok E., Quach L., Datt A., Degenhardt J.D., Panchal A., Seto P., Krakow J.L., Wall R., Hillier B.J. (2022). Regression of EGFR positive established solid tumors in mice with the conditionally active T cell engager TAK-186. J. Immunother. Cancer.

[88] Gong N., Han X., Xue L., Billingsley M.M., Huang X., El-Mayta R., Qin J., Sheppard N.C., June C.H., Mitchell M.J. (2024). Small-molecule-mediated control of the anti-tumour activity and off-tumour toxicity of a supramolecular bispecific T cell engager. Nat. Biomed. Eng..

[89] Nenclares P., Rullan A., Tam K., Dunn L.A., St John M., Harrington K.J. (2022). Introducing Checkpoint Inhibitors Into the Curative Setting of Head and Neck Cancers: Lessons Learned, Future Considerations. Am. Soc. Clin. Oncol. Educ. Book.

[90] Huber L., Kohler C., Lammert A., Scherl C., Ludwig S., Affolter A., Rotter N., Jungbauer F. (2025). Real-World Outcomes of Immune Checkpoint Inhibitors in Head and Neck Squamous Cell Carcinoma: Analyzing Patient-Specific Factors Influencing Survival and Response Rate. Cancer Rep..

[91] Albayrak G., Wan P.K.T., Fisher K., Seymour L.W. (2025). T cell engagers: Expanding horizons in oncology and beyond. Br. J. Cancer.

[92] Laoubi L., Gobbini E., Duplouye P., Hubert M., Caux C., Bendriss-Vermare N., Valladeau-Guilemond J., Voissière A. (2025). Dendritic cells: The central partner for cancer immunity. Oncoimmunology.

[93] Chen S., Zhu H., Jounaidi Y. (2024). Comprehensive snapshots of natural killer cells functions, signaling, molecular mechanisms and clinical utilization. Signal Transduct. Target. Ther..

[94] Shapir Itai Y., Barboy O., Salomon R., Bercovich A., Xie K., Winter E., Shami T., Porat Z., Erez N., Tanay A. (2024). Bispecific dendritic-T cell engager potentiates anti-tumor immunity. Cell.

[95] Appleton E., Chiocca E.A., Ungerechts G., Melcher A., Vile R. (2025). Oncolytic viruses as anticancer agents: Clinical progress and remaining challenges. Lancet.

[96] Chen C., Cillis J., Deshpande S., Park A.K., Valencia H., Kim S.I., Lu J., Vashi Y., Yang A., Zhang Z. (2025). Oncolytic Virotherapy in Solid Tumors: A Current Review. BioDrugs.

[97] Zhou X., Hu S., Wang X. (2025). Recent advances in oncolytic virus combined immunotherapy in tumor treatment. Genes Dis..

[98] Zhang J., Yu S., Peng Q., Wang P., Fang L. (2024). Emerging mechanisms and implications of cGAS-STING signaling in cancer immunotherapy strategies. Cancer Biol. Med..

[99] Paquette B., Oweida A. (2024). Combination of radiotherapy and immunotherapy in duality with the protumoral action of radiation. Cancer Radiother. J. Soc. Fr. Radiother. Oncol..

[100] Zandberg D.P., Menk A.V., Velez M., Normolle D., DePeaux K., Liu A., Ferris R.L., Delgoffe G.M. (2021). Tumor hypoxia is associated with resistance to PD-1 blockade in squamous cell carcinoma of the head and neck. J. Immunother. Cancer.

[101] Wildsmith S., Ye J., Franks A., Melillo G., Armstrong J., Whiteley J., Schnittker K., Lian F., Roland B., Sabalos C. (2022). Association of PD-L1 Expression on Tumor and Immune Cells with Survival in Recurrent or Metastatic Head and Neck Squamous Cell Carcinoma and Assay Validation. Cancer Res. Commun..

[102] McCoon P., Wang Y., Lai Z., Zhang Q., Li W., Wildsmith S., Morsli N., Raja R., Holoweckyj N., Walker J. (2025). Mutational Landscape of Recurrent/Metastatic Head and Neck Squamous Cell Carcinoma and Association with Immune Checkpoint Inhibitor Outcomes. Clin. Cancer Res..

[103] Serafini M.S., Cavalieri S., Licitra L., Pistore F., Lenoci D., Canevari S., Airoldi M., Cossu Rocca M., Strojan P., Grasic Kuhar C. (2024). Association of a gene-expression subtype to outcome and treatment response in patients with recurrent/metastatic head and neck squamous cell carcinoma treated with nivolumab. J. Immunother. Cancer.

[104] Cannataro V.L., Gaffney S.G., Sasaki T., Issaeva N., Grewal N.K.S., Grandis J.R., Yarbrough W.G., Burtness B., Anderson K.S., Townsend J.P. (2019). APOBEC-induced mutations and their cancer effect size in head and neck squamous cell carcinoma. Oncogene.

[105] Ruffin A.T., Cillo A.R., Tabib T., Liu A., Onkar S., Kunning S.R., Lampenfeld C., Atiya H.I., Abecassis I., Kürten C.H.L. (2021). B cell signatures and tertiary lymphoid structures contribute to outcome in head and neck squamous cell carcinoma. Nat. Commun..

[106] Helmink B.A., Reddy S.M., Gao J., Zhang S., Basar R., Thakur R., Yizhak K., Sade-Feldman M., Blando J., Han G. (2020). B cells and tertiary lymphoid structures promote immunotherapy response. Nature.

[107] Vanhersecke L., Brunet M., Guégan J.P., Rey C., Bougouin A., Cousin S., Le Moulec S., Besse B., Loriot Y., Larroquette M. (2021). Mature tertiary lymphoid structures predict immune checkpoint inhibitor efficacy in solid tumors independently of PD-L1 expression. Nat. Cancer..

[108] Kulasinghe A., Taheri T., O’Byrne K., Hughes B.G.M., Kenny L., Punyadeera C. (2020). Highly Multiplexed Digital Spatial Profiling of the Tumor Microenvironment of Head and Neck Squamous Cell Carcinoma Patients. Front. Oncol..

[109] Helou V., Abdul-Rahman N.H., Loo S.K., Gao S.J., Zevallos J.P., Zandberg D.P., Spector M.E., Skinner H.D., Ferris R.L., Contrera K.J. (2025). Circulating tumor DNA in Non-Viral head and neck squamous cell Carcinoma: A systematic review and Meta-Analysis. Oral Oncol..

[110] Chera B.S., Kumar S., Beaty B.T., Marron D., Jefferys S., Green R., Goldman E.C., Amdur R., Sheets N., Dagan R. (2019). Rapid Clearance Profile of Plasma Circulating Tumor HPV Type 16 DNA during Chemoradiotherapy Correlates with Disease Control in HPV-Associated Oropharyngeal Cancer. Clin. Cancer Res..

[111] Rettig E.M., Faden D.L., Sandhu S., Wong K., Faquin W.C., Warinner C., Stephens P., Kumar S., Kuperwasser C., Richmon J.D. (2022). Detection of circulating tumor human papillomavirus DNA before diagnosis of HPV-positive head and neck cancer. Int. J. Cancer..

[112] Strati A., Koutsodontis G., Papaxoinis G., Angelidis I., Zavridou M., Economopoulou P., Kotsantis I., Avgeris M., Mazel M., Perisanidis C. (2017). Prognostic significance of PD-L1 expression on circulating tumor cells in patients with head and neck squamous cell carcinoma. Ann. Oncol..

[113] Economopoulou P., Perisanidis C., Giotakis E.I., Psyrri A. (2016). The emerging role of immunotherapy in head and neck squamous cell carcinoma (HNSCC): Anti-tumor immunity and clinical applications. Ann. Transl. Med..

[114] Oliveira G., Egloff A.M., Afeyan A.B., Wolff J.O., Zeng Z., Chernock R.D., Zhou L., Messier C., Lizotte P., Pfaff K.L. (2023). Preexisting tumor-resident T cells with cytotoxic potential associate with response to neoadjuvant anti—PD-1 in head and neck cancer. Sci. Immunol..

[115] Oguejiofor K., Galletta-Williams H., Dovedi S.J., Roberts D.L., Stern P.L., West C.M.L. (2017). Distinct patterns of infiltrating CD8+ T cells in HPV+ and CD68 macrophages in HPV- oropharyngeal squamous cell carcinomas are associated with better clinical outcome but PD-L1 expression is not prognostic. Oncotarget.

[116] Lyford-Pike S., Peng S., Young G.D., Taube J.M., Westra W.H., Akpeng B., Bruno T.C., Richmon J.D., Wang H., Bishop J.A. (2013). Evidence for a role of the PD-1:PD-L1 pathway in immune resistance of HPV-associated head and neck squamous cell carcinoma. Cancer Res..

[117] Tosi A., Parisatto B., Menegaldo A., Spinato G., Guido M., Del Mistro A., Bussani R., Zanconati F., Tofanelli M., Tirelli G. (2022). The immune microenvironment of HPV-positive and HPV-negative oropharyngeal squamous cell carcinoma: A multiparametric quantitative and spatial analysis unveils a rationale to target treatment-naïve tumors with immune checkpoint inhibitors. J. Exp. Clin. Cancer Res..

[118] Xie X., Dong J., Bi J., Zhai J., Yan B., Li Y., Wang J., Zhu Y. (2024). CRPA1A2: A novel TCR-based T-cell engager targeting MAGEA1-positive solid tumors. J. Clin. Oncol..

[119] Maloney M.F., Caushi J.X., Cullen L.A., Andrews A.R., Ford A.C., Gizzi A.S., Souza C., Stokes K.L., Dobrev V.S., Krapfl K. (2026). Preclinical characterization of CLSP-1025, a first-in-class, mutation-specific T-cell engager targeting a neoantigen derived from a common p53 mutation. Clin. Cancer Res..

[120] Johnson M., El-Khoueiry A., Hafez N., Lakhani N., Mamdani H., Rodon J., Sanborn R.E., Garcia-Corbacho J., Boni V., Stroh M. (2021). Phase I, First-in-Human Study of the Probody Therapeutic CX-2029 in Adults with Advanced Solid Tumor Malignancies. Clin. Cancer Res..

[121] Boni V., Fidler M.J., Arkenau H.-T., Spira A., Meric-Bernstam F., Uboha N., Sanborn R.E., Sweis R.F., LoRusso P., Nagasaka M. (2022). Praluzatamab Ravtansine, a CD166-Targeting Antibody—Drug Conjugate, in Patients with Advanced Solid Tumors: An Open-Label Phase I/II Trial. Clin. Cancer Res..

